# Machine learning-based assessment of tumor-infiltrating lymphocytes in breast cancer histopathology: a systematic review and evidence-gap analysis of translational readiness

**DOI:** 10.3389/fonc.2026.1908349

**Published:** 2026-10-26

**Authors:** Gauhar Dunenova, Aidos Sarsembayev, Ainash Oshibayeva, Saltanat Kyrykbayeva, Zinat Abdrakhmanova, Balnur Omarova, Alexandr Ivankov, Aliya Ualiyeva, Balzhan Zhakypova, Zhanar Zhagiparova, Gulnaz Nuskabayeva, Meruyert Suleimenova, Natalya Glushkova

**Affiliations:** 1 Khoja Akhmet Yassawi International Kazakh-Turkish University, Turkestan, Kazakhstan; 2 School of Digital Technologies, Almaty Management University, Almaty, Kazakhstan; 3 Office of the Vice Rector for Science and Strategic Development, Khoja Akhmet Yassawi International Kazakh-Turkish University, Turkestan, Kazakhstan; 4 Center for Strategic Development, Ranking, and Quality, Khoja Akhmet Yassawi International Kazakh-Turkish University, Turkestan, Kazakhstan; 5 Health Research Institute, Al-Farabi Kazakh National University, Almaty, Kazakhstan; 6 Faculty of Medicine, Khoja Akhmet Yassawi International Kazakh-Turkish University, Turkestan, Kazakhstan; 7 Department of Epidemiology, Biostatistics and Evidence-Based Medicine, Al-Farabi Kazakh National University, Almaty, Kazakhstan; 8 Faculty of Medicine and Health Care, Al-Farabi Kazakh National University, Almaty, Kazakhstan; 9 Postgraduate Faculty of Medicine, Khoja Akhmet Yassawi International Kazakh-Turkish University, Turkestan, Kazakhstan

**Keywords:** artificial intelligence, breast cancer, computational pathology, evidence-gap analysis, machine learning, systematic review, translational readiness, tumor-infiltrating lymphocytes

## Abstract

**Background:**

Machine learning-based assessment of tumor-infiltrating lymphocytes (TILs) has been proposed to improve the reproducibility and clinical utility of immune-contexture evaluation in breast cancer histopathology. However, existing studies differ substantially in task definition, annotation strategy, model architecture, validation design, and clinical endpoint. This systematic review and evidence-gap analysis aimed to map the translational readiness of TIL-related machine learning studies in breast cancer histopathology and to identify cluster-specific methodological gaps.

**Methods:**

A systematic search of PubMed, Web of Science, Scopus, ScienceDirect, and Google Scholar was conducted up to 20 April 2026, supplemented by backward citation tracing and a targeted Google search of the 30 most relevant additional results. Eligible studies included breast cancer histopathology-based machine learning, deep learning, artificial intelligence, or computational image-analysis studies evaluating TILs or TIL-related immune-cell features. Studies were grouped by principal application into detection/classification, outcome prognostication, and treatment-response prediction. Extracted variables included data source, specimen type, model family, annotation strategy, validation design, clinical comparator use, calibration, decision-curve analysis, incremental value, reproducibility, and methodological challenges. Because of heterogeneity in endpoints and performance metrics, synthesis was descriptive and evidence-map based.

**Results:**

The primary evidence map included 51 histopathology-image studies: 19 detection/classification, 18 outcome prognostication, and 14 treatment-response prediction studies. External validation was reported in 31.6% of detection/classification studies, 61.1% of prognostication studies, and 57.1% of treatment-response prediction studies. Clinical comparator reporting increased across the same continuum, from 63.2% to 94.4% and 92.9%, respectively. However, calibration was reported in only one prognostication study (5.6%) and in no detection/classification or treatment-response prediction studies. Decision-curve analysis was similarly limited to one prognostication study. Class imbalance was near-universal (94.7–100% across clusters). Scanner/stain robustness was addressed in a majority of studies (63.2–83.3%), but fairness or subgroup analyses remained inconsistently reported (27.8–57.1%).

**Conclusions:**

TIL machine learning in breast cancer is technically active but clinically under-validated. Future studies should move beyond model development toward externally validated, calibrated, clinically benchmarked, reproducible, and decision-relevant evidence, using cluster-specific planning standards for detection, prognostication, and treatment-response prediction.

**Systematic review registration:**

https://www.crd.york.ac.uk/PROSPERO/view/CRD42024574566, identifier CRD42024574566.

## 1 Introduction

Breast cancer remains one of the most significant health burdens worldwide, accounting for a substantial proportion of cancer-related morbidity and mortality. According to the WHO, it was the most commonly diagnosed cancer among women in 157 out of 185 countries in 2022 and the leading cause of cancer death in this population (1). The age-standardized incidence rate of breast cancer is expected to increase from 45.86 cases per 100,000 women in 2019 to 48.09 cases per 100,000 women in 2030 (2). Advances in early detection and treatment have improved survival rates, but the disease still poses considerable challenges, particularly in terms of prognosis, treatment response variability, regional disparities and mental health challenges (3). It is predicted that in high human development index countries, 1 in 71 women will die from breast cancer, whereas in low human development index countries, 1 in 48 women will die from breast cancer in their lifetime (1).

Tumor-infiltrating lymphocytes (TIL) are a crucial component of the immune response against tumors, and their presence in breast cancer is associated with improved clinical outcomes (4). A systematic review of TILs’ predictive value in breast cancer indicates that a high TIL score can predict a better response to neoadjuvant chemotherapy (NAC) in most breast cancer types (5). However, the presence of PD-1+ TIL subtype is associated with poor prognosis in breast cancer (6). The assessment of TILs offers several advantages, including potential predictive and prognostic value, which can aid in tailoring personalized treatment strategies (7). Nevertheless, traditional methods of TIL assessment, such as manual scoring by pathologists, are subjective and time-consuming (8), leading to variability in results and limiting their clinical utility despite the availability of evaluation guidelines by an International TILs Working Group (9). The role of TILs in breast cancer treatment decisions remains uncertain due to insufficient data to guide specific therapy choices and treatment management decisions, as well as the lack of standardized uniform procedures for their isolation and characterization (10, 11).

The Fourth Industrial Revolution has introduced Machine Learning (ML) and artificial intelligence (AI) into data science and analytics (12). The advancements in ML have transformed the assessment of tumor-infiltrating lymphocytes (TILs) in whole slide images (WSIs), enabling more objective, reproducible, and efficient analyses through computer-assisted pathology (CAP) tools (13). ML-based approaches have shown promise in accurately identifying and quantifying TILs, potentially enhancing prognostic and predictive models. These technological innovations could lead to more standardized and reliable TIL assessments, contributing to better patient management and outcomes. An effort has also been made to evaluate publicly available breast cancer datasets to provide foundational data to train and validate ML-based models (13).

Despite these advancements, several challenges remain in implementing ML-based TIL assessment in clinical practice. A report by the International Immuno-Oncology Biomarker Working Group on Breast Cancer in 2023 identifies four major challenges in ML-based TIL assessment: technical slide challenges, ML and image analysis challenges, data challenges, and validation challenges (14). To effectively train ML-based models, a large amount of accurately annotated data is required (15). The predictive value of TILs is well established in human epidermal growth factor receptor 2 positive (HER2-positive) and triple-negative breast cancer (TNBC) subtypes, where increased TIL levels are associated with better responses to neoadjuvant chemotherapy and improved clinical outcomes (16). However, in other breast cancer subtypes, the predictive significance of TILs is less consistent. What remains unclear is whether these models have progressed from technical feasibility to clinically translatable evidence. This will help guide future research and improve the clinical applicability of these innovative techniques.

Accordingly, this systematic review and evidence-gap analysis aimed to map the translational and methodological readiness of machine learning-based studies of tumor-infiltrating lymphocytes in breast cancer histopathology. Specifically, this review addressed the following question: What are the main translational evidence gaps and methodological challenges in machine learning-based analysis of tumor-infiltrating lymphocytes in breast cancer histopathology, and how do these gaps differ across detection/classification, outcome prognostication, and treatment-response prediction studies?

This review differs from prior summaries of automated TIL scoring in two respects. First, we group studies by clinical application because the evidentiary standard expected at each stage differs by task, as our findings demonstrate. Second, we use an evidence-gap-analysis framework to produce three cluster-specific planning checklists, intended as a prospective resource for investigators designing future TIL-ML studies.

## 2 Materials and methods

### 2.1 Study design and reporting framework

We conducted a systematic review and evidence-gap analysis of machine learning-based studies evaluating tumor-infiltrating lymphocytes in breast cancer. Two reviewers (G.D. and M.S.) independently conducted all stages of the systematic review, including development of the search strategy, record screening, full-text assessment, data extraction, and eligibility decisions, with disagreements resolved through discussion with the third reviewer (N.G.). The reporting followed the Preferred Reporting Items for Systematic Reviews and Meta-Analyses (PRISMA 2020) guidelines (17). Because the included studies differed substantially in their intended clinical task, unit of analysis, annotation strategy, model architecture, validation design, and performance metrics, a meta-analysis was not planned.

The protocol was registered prospectively in PROSPERO under registration number CRD42024574566. The review was based entirely on published or publicly available reports and did not involve individual participant data; therefore, additional patient consent was not required.

### 2.2 Information sources and search strategy

A systematic literature search was performed in PubMed, Web of Science, Scopus, ScienceDirect, and Google Scholar. The search was completed on 20 April 2026. The search strategy combined terms related to breast cancer, tumor-infiltrating lymphocytes, artificial intelligence or machine learning, digital pathology or histopathology, and clinically relevant endpoints. The core search concepts were: breast cancer or breast carcinoma; tumor-infiltrating lymphocytes or TILs; machine learning, deep learning, artificial intelligence, neural networks, or computational pathology. Boolean operators were adapted to the syntax of each database. Reference lists of included studies and relevant reviews were also screened to identify additional eligible studies. Example PubMed search string: ((“Breast Neoplasms”[Mesh] OR breast cancer[tiab] OR breast carcinoma[tiab] OR mammary cancer[tiab])) AND ((“Tumor-Infiltrating Lymphocytes”[Mesh] OR tumor infiltrating lymphocytes[tiab] OR TIL[tiab] OR TILs[tiab] OR stromal TIL[tiab] OR intratumoral TIL[tiab])) AND ((“Machine Learning”[Mesh] OR “Artificial Intelligence”[Mesh] OR “Deep Learning”[Mesh] OR machine learning[tiab] OR deep learning[tiab] OR artificial intelligence[tiab] OR neural network*[tiab] OR convolutional neural network*[tiab] OR CNN[tiab] OR computational pathology[tiab])).

For Google Scholar, screening was restricted to the titles only. The database-specific search strings are provided in **Supplementary Table 1**.

During revision, the titles of the extracted records were rechecked against the prespecified eligibility criteria. We also performed backward citation tracing of the reference lists of the 36 studies included in the original review. Potentially relevant reports identified through title rechecking, backward citation tracing, and the supplementary Google search were deduplicated against the original search library and assessed at full text using the original eligibility criteria. No changes were made to the review question, population, eligible histopathology modalities, machine-learning requirements, application clusters, outcomes, or synthesis methods.

### 2.3 Eligibility criteria

Studies were eligible if they met all of the following criteria: they included patients with breast cancer or breast cancer tissue samples; evaluated tumor-infiltrating lymphocytes or TIL-related immune-cell features; used machine learning, deep learning, artificial intelligence, or computational image-analysis methods; used histopathology images derived from hematoxylin and eosin-stained or immunohistochemistry-stained tissue, including whole-slide images, tissue microarrays, biopsies, or surgical specimens; and reported at least one methodological, technical, diagnostic, prognostic, predictive, or validation-relevant outcome.

Eligible study designs included original research articles, full conference papers, theses, and preprints if sufficient methodological information was available for extraction. Reviews, editorials, letters, commentaries, case reports, meeting abstracts without sufficient technical detail, animal studies, non-breast-cancer studies, and studies relying only on manual TIL scoring without computational or machine learning-based assessment were excluded.

Because the field includes several cross-modal studies in which TIL status is predicted from non-histopathology data or combined with molecular data, such studies were not treated as part of the primary histopathology-image evidence map and were also excluded.

### 2.4 Study selection

All records retrieved from the search were imported into a reference-management database, and duplicates were removed before screening. Titles and abstracts were screened independently by two reviewers against the eligibility criteria. Records judged potentially eligible by either reviewer proceeded to full-text review. Full-text articles were then assessed independently by two reviewers, and reasons for exclusion were documented. Disagreements were resolved by discussion and, when necessary, adjudication by a third reviewer.

The study-selection process was summarized using a PRISMA flow diagram. A thorough search across PubMed, Web of Science, ScienceDirect, Scopus, and Google Scholar databases yielded 346 records. Initial screening reduced this to 211 non-duplicative records, from which 103 full-text articles underwent evaluation, and 36 studies were included in the initial step. Eleven articles were included in the detection/classification cluster, fifteen articles were included in the outcome prognostication cluster, and ten articles were included in the treatment-response prediction cluster. Two studies that evaluated ML-based TIL assessment using ultrasound images were excluded (18, 19), as was one study focusing on immune-related TIL-associated hub genes (20).

Following the initial study selection, backward citation searching was conducted using the reference lists of the 36 included studies, comprising 1,647 cited references. A supplementary Google Scholar search was performed, restricted to the 30 most relevant results. These additional searches identified 17 potentially relevant reports. After removal of one duplicate publication version, 16 full-text reports were assessed for eligibility. One report was excluded because it used molecular or transcriptomic data without eligible histopathology-image analysis (21), and 15 additional studies were included.

The final analytic sample therefore comprised 51 studies: 19 in the detection/classification cluster, 18 in the outcome-prognostication cluster, and 14 in the treatment-response prediction cluster. The complete study-selection process, including reasons for exclusion, is presented in **Figure 1** (17).

**Figure 1:**
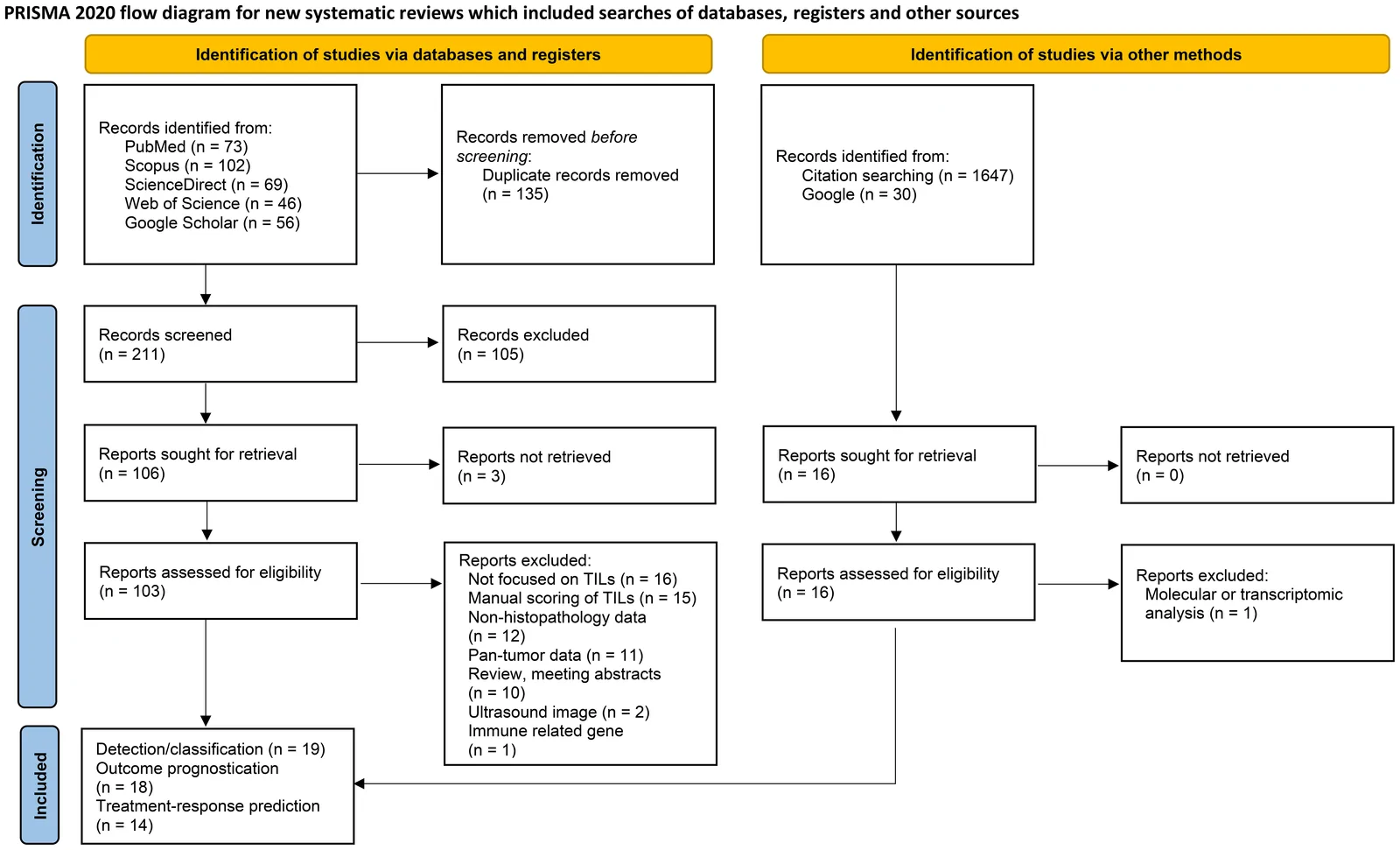
PRISMA flow diagram of study selection process.

### 2.5 Classification of studies into application clusters

Included studies were classified according to their principal application rather than only by model architecture. Three application clusters were defined *a priori*.

Cluster 1 included detection or classification studies, defined as studies whose primary aim was to identify, segment, classify, localize, or quantify TILs or related immune cells on histopathology images. These studies typically used pixel-level, nucleus-level, patch-level, or region-level annotations and commonly reported technical performance metrics such as Dice coefficient, F1 score, precision, recall, accuracy, or area under the receiver operating characteristic curve.

Cluster 2 included outcome prognostication studies, defined as studies evaluating whether machine learning-derived TIL, immune-cell, or tumor microenvironment features were associated with survival or recurrence outcomes. These studies typically reported patient-level endpoints such as overall survival, disease-free survival, recurrence-free survival, invasive disease-free survival, progression-free interval, or disease-specific survival, and commonly used Cox regression, C-index, hazard ratios, or related survival metrics.

Cluster 3 included treatment-response prediction studies, defined as studies using TIL-related or histopathology-derived machine learning features to predict response to neoadjuvant or systemic therapy. These studies most commonly evaluated pathological complete response or residual disease after neoadjuvant chemotherapy and reported metrics such as area under the curve, odds ratios, accuracy, sensitivity, specificity, or response-stratified survival outcomes.

### 2.6 Data extraction

Data were extracted using a structured study-level extraction template developed specifically for this review. The extraction form was piloted on a subset of studies and refined before full extraction. For each included study, we extracted bibliographic details, publication year, country, setting, study design, breast cancer subtype, specimen type and timing, staining modality, image source, number of patients and images, unit of analysis, dataset type, and use of named public or challenge cohorts. When studies used multiple datasets, development, internal-validation, and external-validation cohorts were recorded separately where sufficient information was available.

Technical variables included the primary application cluster, machine learning task, model family, use of classical machine learning, convolutional neural networks, U-Net or encoder-decoder segmentation models, object detection models, multiple-instance learning, transformer or foundation models, self-supervised or histology-specific pretraining, and multimodal integration. Annotation variables included the level of annotation, number and type of annotators, whether pathologist consensus was used, whether interobserver agreement was reported, whether stromal and intratumoral TILs were distinguished, and whether spatial TIL features or broader tumor microenvironment features were assessed.

Validation and clinical-readiness variables included internal validation, cross-validation, external validation, multicenter design, confirmed non-overlap between training and validation data, independent test set, calibration reporting, decision-curve analysis, clinical comparator, incremental value over clinicopathological models, confidence intervals for the primary metric, code availability, data availability, model-weight availability, and public benchmark use. When public datasets were used, data availability was classified as “available.”

Methodological challenge variables were coded using predefined criteria. These included class imbalance, pathologist ground-truth variability, small sample concern, single-center concern, endpoint heterogeneity, treatment heterogeneity, scanner or staining variability, potential data leakage or non-independent validation, limited reproducibility, and absence of fairness or subgroup analysis. Each variable was coded as yes, no, unclear, or not applicable, depending on the information available in the full text.

During the revision process, we added information on explainability, reproducibility, and deployment, and summarized the potential clinical applicability of each study. Data extraction was performed independently by two reviewers, and disagreements were resolved through discussion and, when necessary, consultation with a third reviewer.

### 2.7 Evidence-gap framework

The evidence-gap framework was designed to evaluate translational readiness across clusters. The framework crossed each application cluster with a set of methodological and clinical-readiness indicators: external validation, confirmed independent validation, calibration, decision-curve analysis, clinical comparator, incremental value, public benchmark use, code availability, data availability, model-weight availability, and fairness or subgroup analysis.

For each cluster and indicator, we calculated the number and percentage of studies reporting the indicator. Evidence maturity was categorized descriptively as follows: major gap when fewer than 25% of studies reported the indicator; moderate gap when 25% to less than 50% reported it; partial evidence when 50% to less than 75% reported it; and relatively mature evidence when 75% or more reported it. These thresholds were used for evidence mapping and interpretation only and were not intended as formal statistical cut-points.

The resulting evidence-gap matrix was used to generate heatmaps and summary tables showing which areas of the TIL machine learning literature were comparatively mature and which remained insufficiently developed for clinical translation.

### 2.8 Reporting completeness appraisal

The methodological quality of included studies were evaluated using two complementary, guideline-based instruments tailored to prediction modeling and AI research: the Transparent Reporting of a multivariable prediction model for Individual Prognosis Or Diagnosis–Artificial Intelligence extension (TRIPOD+AI) and the Prediction model Risk Of Bias ASsessment Tool (PROBAST).

The TRIPOD+AI checklist was applied to assess the completeness and transparency of reporting in each included study (22, 23). This tool extends the original TRIPOD statement to address specific considerations relevant to ML and AI-based prediction models, including data preprocessing, model development, validation strategies, and reproducibility. Each study was evaluated across all applicable TRIPOD+AI items, which encompass domains related to title and abstract, background and objectives, data sources and participants, outcome definition, predictor specification, model development, performance reporting, interpretation, and availability of **Supplementary Information**. For each item, reporting was classified as “Yes” (reported), “No” (not reported), or “Not applicable” when the item was not relevant to the study design. To summarize overall reporting quality, the number of reported items was calculated as a proportion of all applicable items, excluding those deemed not applicable. Consistent with prior methodological reviews, studies reporting more than 70% of applicable items were classified as having high reporting completeness, whereas those reporting fewer than 70% were classified as having lower reporting completeness. In addition, for descriptive purposes, a continuous quality score was calculated for each study and expressed as the number and percentage of reported items.

Methodological rigor was assessed using the PROBAST tool, which is specifically designed to evaluate the methodological rigor of prediction model studies (22, 24). PROBAST comprises four domains: participants, predictors, outcomes, and analysis. Each domain includes multiple signaling questions that guide judgments regarding potential sources of bias and concerns about applicability. For each signaling question, responses were categorized as “Yes,” “Probably yes,” “Probably no,” “No,” or “No information.” Domain-level risk of bias was then determined according to PROBAST guidance: a domain was rated as low risk if all signaling questions were answered affirmatively; as high risk if one or more questions indicated potential bias (i.e., “No” or “Probably no”); and as unclear if insufficient information was provided. An overall risk of bias judgment was subsequently assigned to each study. Studies were classified as having high overall risk of bias if at least one domain was rated as high risk, moderate (or unclear) risk if no domains were high risk but at least one domain was unclear, and low risk if all domains were rated as low risk.

To facilitate comparative interpretation and identify patterns across studies, visual summaries were generated for both tools. For TRIPOD+AI, item-level reporting frequencies were aggregated across studies and presented as stacked bar plots, illustrating the proportion of studies that reported, did not report, or were not applicable for each checklist item. For PROBAST, domain-level distributions of risk of bias were visualized using horizontal stacked bar charts, displaying the proportion of studies classified as low, high, or unclear risk within each domain, as well as overall risk of bias. Assessments were conducted independently by two reviewers, with discrepancies resolved through discussion.

### 2.9 Data synthesis and statistical analysis

The synthesis was descriptive, and evidence-mapping based. Study characteristics were summarized overall and by application cluster using frequencies and percentages for categorical variables and medians or ranges for continuous variables where appropriate. No inferential comparisons between clusters were performed because the purpose was to map evidence gaps rather than to test hypotheses, and because cluster denominators were small.

Performance metrics were summarized narratively within each cluster because metrics were not comparable across tasks. Detection and segmentation studies reported technical metrics such as Dice coefficient, F1 score, precision, recall, and AUC. Prognostic studies reported hazard ratios, C-indices, survival-model performance, or associations with survival outcomes. Treatment-response studies reported AUC, odds ratios, accuracy, or pathological complete response-related performance. Because of this heterogeneity in endpoints, units of analysis, and performance metrics, quantitative pooling was not appropriate.

Evidence-gap visualizations were generated from the completed extraction dataset. All analyses were conducted in R version 4.5.1 (2025-06-13). The final extraction dataset was stored in Microsoft Excel format.

Because eligible studies reported non-comparable outcome metrics by task, quantitative pooling was not appropriate, and synthesis was descriptive, and evidence-map based.

## 3 Results

The primary evidence map included 51 histopathology-image machine learning studies, comprising 19 detection/classification studies (25–43), 18 outcome-prognostication studies (44–61), and 14 treatment-response prediction studies (62–75) as detailed in **Supplementary Table 2**.

The distribution of study characteristics demonstrated a general, although less pronounced, translational gradient across the three application clusters (**Table 1**). Detection/classification studies remained more technically oriented, but the revised evidence base showed greater methodological maturity than the initial analysis: 6 of 19 studies used multicenter data (31.6%), 6 reported external validation (31.6%), and 12 included a clinical or pathologist comparator (63.2%). Public, challenge, or mixed public–private datasets were also most common in this cluster, occurring in 12 studies (63.2%), reflecting the increasing use of shared resources for model development and benchmarking.

**Table 1:** Study characteristics by application cluster.

Application cluster	Studies, n	Multi-center, n (%)	Public/private mixed or public data, n (%)	External validation, n (%)	Clinical comparator, n (%)	Calibration reported, n (%)	Decision-curve analysis, n (%)	Code available, n (%)
Detection/classification	19	6 (31.6%)	12 (63.2%)	6 (31.6%)	12 (63.2%)	0 (0.0%)	0 (0.0%)	9 (47.4%)
Outcome prognostication	18	11 (61.1%)	10 (55.6%)	11 (61.1%)	17 (94.4%)	1 (5.6%)	1 (5.6%)	7 (38.9%)
Treatment-response prediction	14	10 (71.4%)	1 (7.1%)	8 (57.1%)	13 (92.9%)	0 (0.0%)	0 (0.0%)	6 (42.9%)

Clinically anchored designs were more frequent among outcome-prognostication and treatment-response prediction studies. Multicenter data were used in 11 of 18 prognostication studies (61.1%) and 10 of 14 treatment-response studies (71.4%). External validation was reported in 11 prognostication studies (61.1%) and 8 treatment-response studies (57.1%). Clinical or pathologist comparators were included in 17 prognostication studies (94.4%) and 13 treatment-response studies (92.9%), compared with 12 detection/classification studies (63.2%). These findings indicate that studies addressing patient-level outcomes more frequently benchmarked machine-learning-derived TIL measures against existing pathological, clinical, or clinicopathological assessments. However, the somewhat lower proportion of external validation in treatment-response studies than in prognostication studies indicates that progression toward direct clinical decision support was not consistently accompanied by stronger independent validation.

Important limitations in clinical readiness remained across all clusters. No detection/classification or treatment-response study reported calibration, while calibration was reported in only one prognostication study (5.6%). Decision-curve analysis was likewise limited to one prognostication study (5.6%) and was absent from the other clusters. Code availability remained inconsistent, ranging from 38.9% in prognostication studies to 47.4% in detection/classification studies and 42.9% in treatment-response studies. Overall, the evidence base showed increasing use of multicenter designs, external validation, and clinical comparators as studies moved toward prognostic and treatment-response applications. Nevertheless, calibration, formal clinical-utility assessment, and reproducibility remained insufficiently addressed, limiting conclusions regarding readiness for clinical implementation.

The evidence-gap heatmap demonstrated substantial heterogeneity in translational readiness across the three TIL-ML application clusters (**Figure 2**). Among applicable studies, external validation was reported in 32% of detection/classification studies, compared with 61% of outcome prognostication studies and 57% of treatment-response prediction studies. Clinical or pathologist comparator reporting was also substantially more frequent in clinically oriented applications, increasing from 63% in detection/classification studies to 94% in outcome prognostication studies and 93% in treatment-response prediction studies. However, indicators of clinical utility remained consistently underdeveloped. Decision-curve analysis or net-benefit assessment was absent from detection/classification and treatment-response prediction studies and was reported in only 6% of outcome prognostication studies. Outcome calibration showed a similarly limited pattern, being absent from detection/classification and treatment-response prediction studies and reported in only 6% of prognostication studies. Incremental-value analysis was reported in 16% of detection/classification studies, 35% of outcome prognostication studies, and 64% of treatment-response prediction studies. Reproducibility indicators were also incomplete. Code availability was reported in 47%, 39%, and 43% of detection/classification, outcome prognostication, and treatment-response prediction studies, respectively. Model weights were available in 33% of applicable detection/classification studies, none of the applicable prognostication studies, and 7% of treatment-response prediction studies. Data availability was comparatively higher in detection/classification studies (63%) and outcome prognostication studies (50%) but remained limited in treatment-response prediction studies (29%). Overall, although prognostication and treatment-response studies more frequently incorporated external validation and clinical benchmarking, translational readiness remained constrained by minimal calibration and decision-curve analysis, limited reporting of incremental clinical value, and incomplete availability of code, model parameters, and reusable datasets.

**Figure 2:**
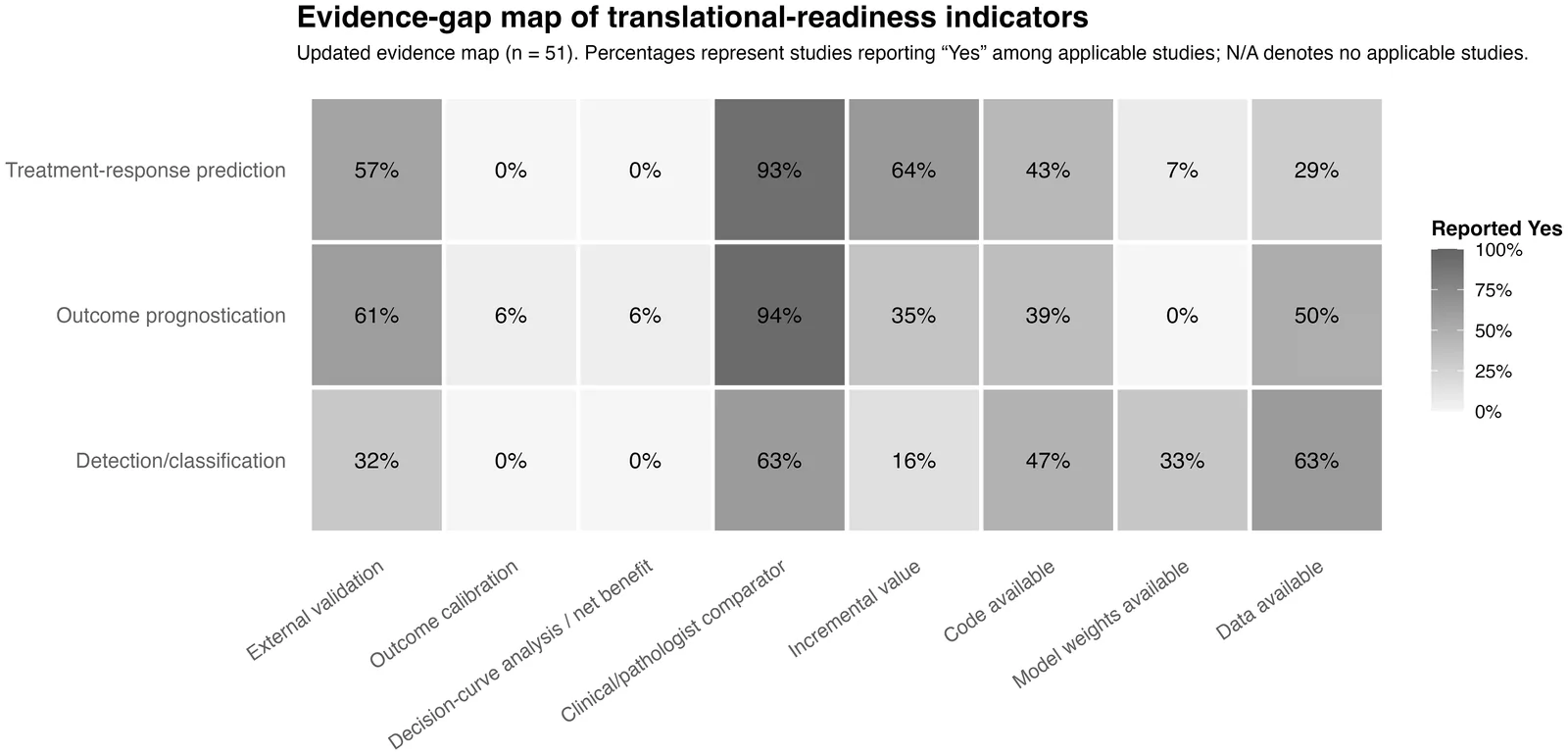
Translational-readiness evidence gaps across TIL-ML application clusters.

Validation and clinical-utility indicators differed substantially across application clusters (**Figure 3**). Among applicable studies, external validation was reported in 32% of detection/classification studies (6/19), compared with 61% of outcome prognostication studies (11/18) and 57% of treatment-response prediction studies (8/14). This pattern suggests that studies targeting more direct clinical decision-making were more likely to assess model performance beyond the development setting. Clinical comparator reporting showed a similar distribution: 63% of detection/classification studies (12/19) compared model outputs against a clinical or pathologist-based comparator, whereas such comparisons were reported in 94% of outcome prognostication studies (17/18) and 93% of treatment-response prediction studies (13/14). Incremental-value analysis was less common overall but increased across the application spectrum, being reported in 16% of detection/classification studies (3/19), 35% of prognostication studies (6/18), and 64% of treatment-response prediction studies (9/14), indicating that treatment-response models were more likely to evaluate whether TIL-derived machine learning features added information beyond existing clinicopathological approaches. In contrast, indicators more directly related to implementation readiness remained poorly developed. Decision-curve analysis or net-benefit assessment was absent in detection/classification and treatment-response prediction studies and was reported in only 6% of prognostication studies (1/18). Outcome calibration showed a similarly limited pattern, with no reporting in detection/classification studies (0/19) or treatment-response prediction studies (0/14), and only one prognostication study reporting calibration (1/18, 6%). Overall, although prognostic and treatment-response studies more frequently incorporated external validation and clinical benchmarking, evidence supporting calibrated prediction, threshold-based clinical decision support, and broader clinical utility remains sparse across the TIL-ML literature.

**Figure 3:**
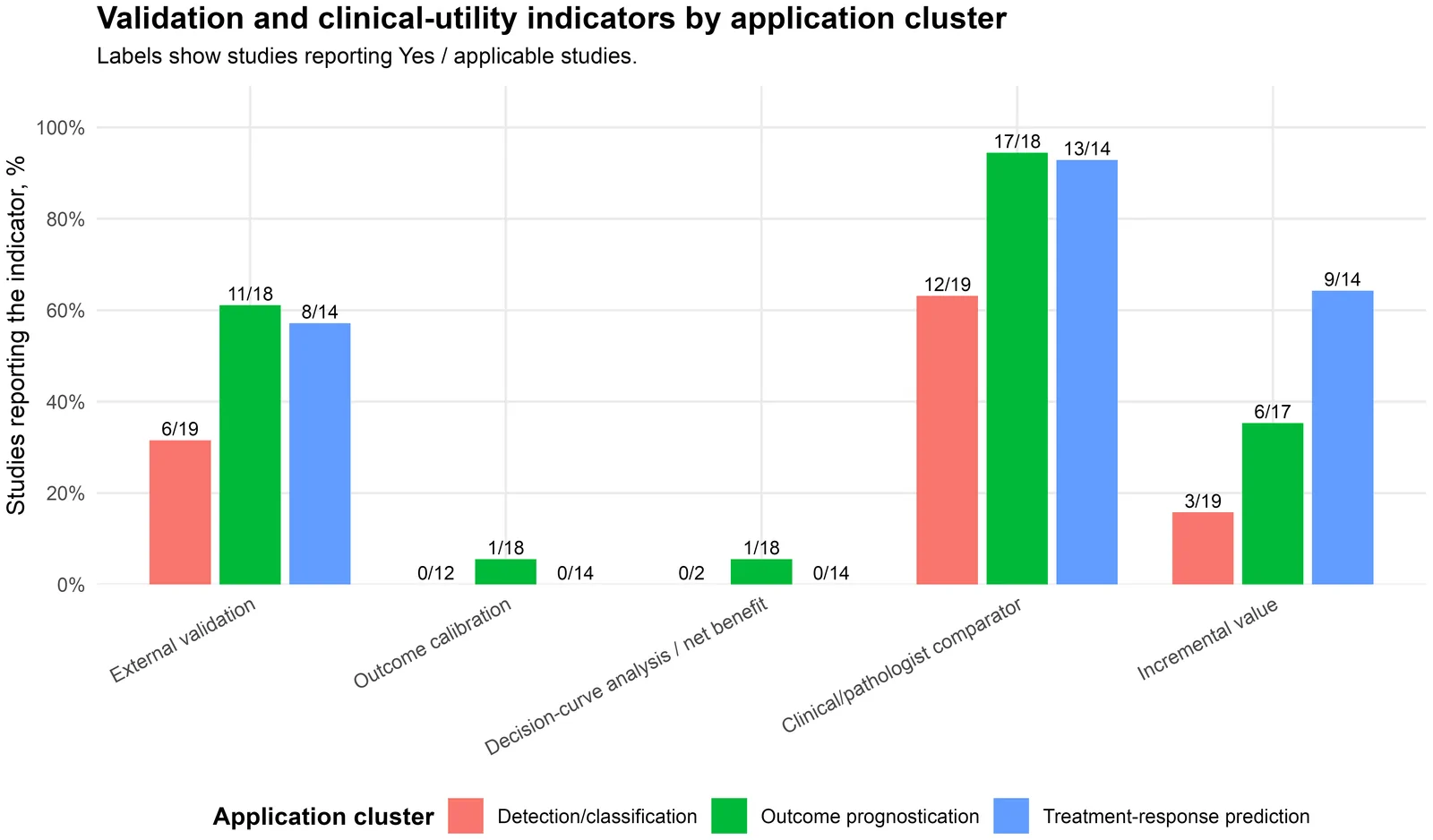
Validation and clinical-utility indicators across TIL-ML application clusters.

The distribution of model families differed across application clusters, indicating that the methodological structure of the field is shaped by the intended clinical task (**Figure 4**). Each study was assigned one dominant model family according to its primary analytical pipeline. Detection/classification studies showed the greatest architectural diversity. Segmentation or object-detection approaches and classical or handcrafted machine learning were the most common, with five studies each, followed by CNN/deep-learning approaches in four studies. The remaining studies used weakly supervised or multiple-instance-learning/attention methods, self-supervised learning, hybrid or multimodal pipelines, foundation or transformer-based approaches, and benchmark or multi-model comparison frameworks, with one study in each category. This diversity reflects the technical breadth of detection and classification tasks, which may involve cell-level identification, tissue or region segmentation, compartment detection, slide-level classification, or automated TIL scoring.

**Figure 4:**
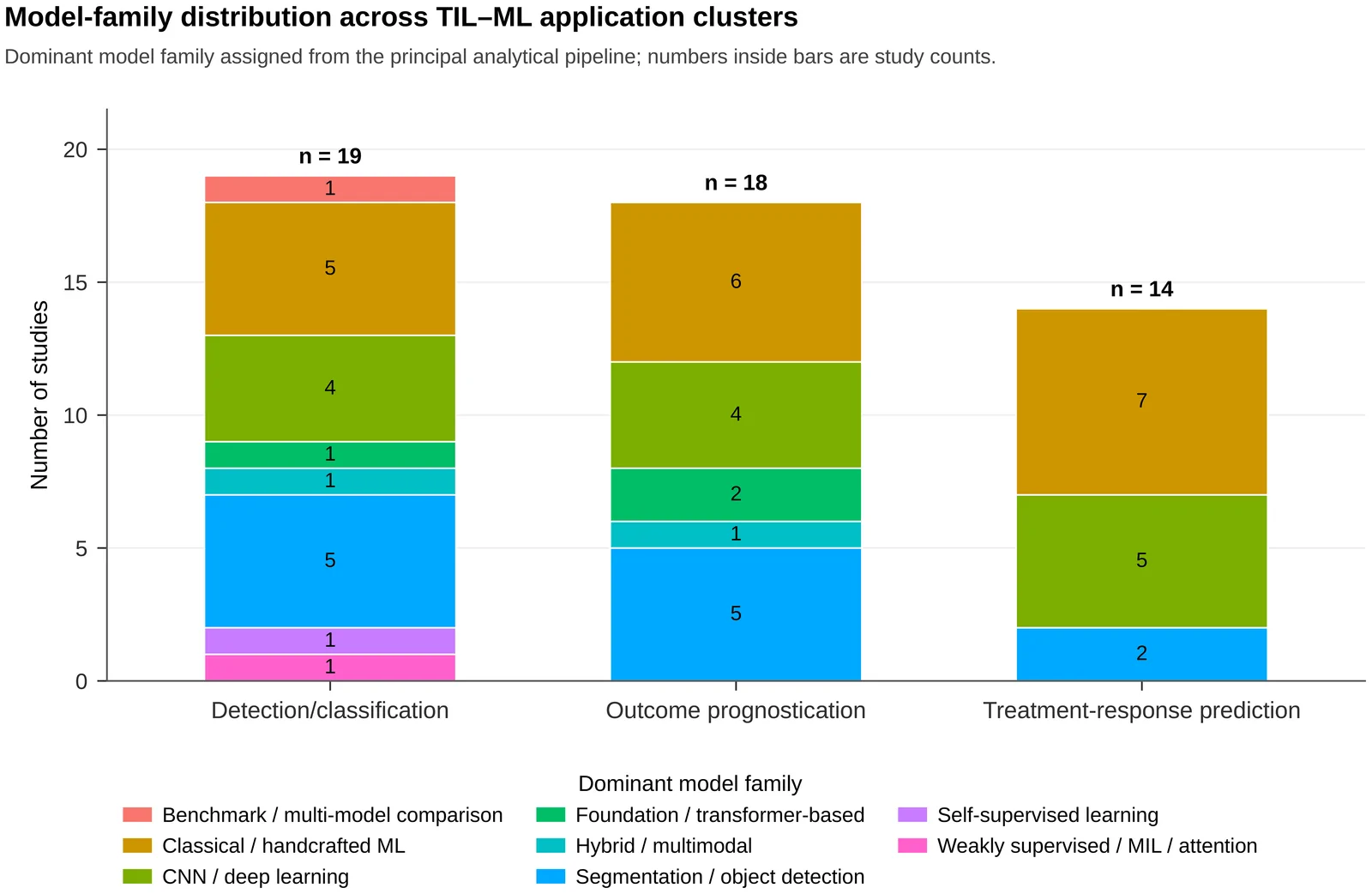
Model-family distribution across TIL-ML application clusters.

Outcome prognostication studies were more concentrated within a smaller number of model families. Classical or handcrafted machine learning was the most frequent approach, used in six studies, followed by segmentation or object-detection methods in five studies and CNN/deep-learning approaches in four studies. Foundation or transformer-based models were used in two studies, while one study used a hybrid or multimodal pipeline. These studies generally converted image-derived TIL, immune-cell, spatial, or tumor-microenvironment features into patient-level prognostic estimates, often using feature aggregation, survival analysis, or conventional machine-learning pipelines.

Treatment-response prediction showed the most concentrated methodological profile. Classical or handcrafted machine learning was used in seven studies, CNN/deep-learning approaches in five studies, and segmentation or object-detection methods in two studies. No treatment-response study was assigned to the benchmark-comparison, hybrid or multimodal, foundation or transformer-based, self-supervised, or weakly supervised/multiple-instance-learning categories. This cluster primarily focused on predicting pathological response or residual disease after neoadjuvant treatment using pretreatment images and associated TIL or tumor-microenvironment features. Overall, the model-family distribution demonstrates substantial task-dependent methodological heterogeneity.

Reuse of public datasets was concentrated within a small number of frequently accessed resources (**Figure 5**). TCGA-BRCA was the most commonly reused cohort, contributing to nine eligible study rows, followed by the TIGER challenge dataset in six studies and BCSS in four. METABRIC appeared in three study rows, whereas ICGC and FinHER were each used in two. This pattern indicates that a substantial proportion of the TIL-ML literature relies on a limited set of shared datasets and benchmark cohorts. Although reuse facilitates methodological comparison and external benchmarking, repeated analyses of the same underlying patient populations do not represent independent clinical evidence and may amplify dataset-specific characteristics. Consequently, the apparent breadth of the evidence base may exceed the true diversity of represented populations, institutions, and clinical settings.

**Figure 5:**
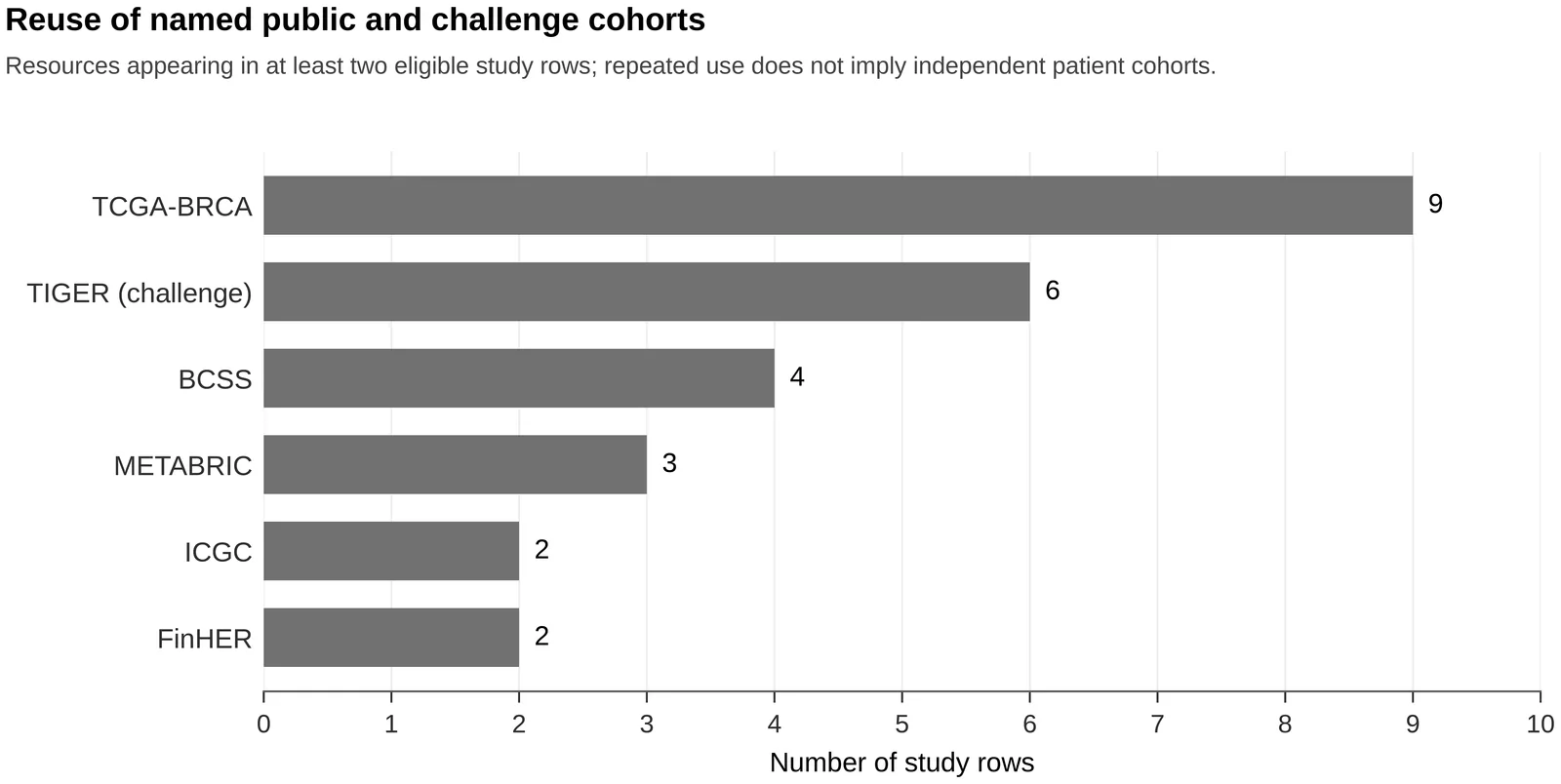
Reuse of public datasets.

Methodological challenges were common across all application clusters, although their distribution and the extent to which they were addressed varied by task (**Figure 6**). Class imbalance was nearly universal, being identified in 18 of 19 detection/classification studies (94.7%) and in all outcome prognostication (18/18, 100%) and treatment-response prediction studies (14/14, 100%). Pathologist variability was also frequently reported in detection/classification studies (15/19, 78.9%) and outcome prognostication studies (14/18, 77.8%), but was less common in treatment-response prediction studies (6/14, 42.9%). This difference may reflect the greater reliance of detection and prognostication models on pathologist-defined TIL labels, tissue compartments, or annotation-derived ground truth, whereas treatment-response studies more often used patient-level clinical outcomes.

**Figure 6:**
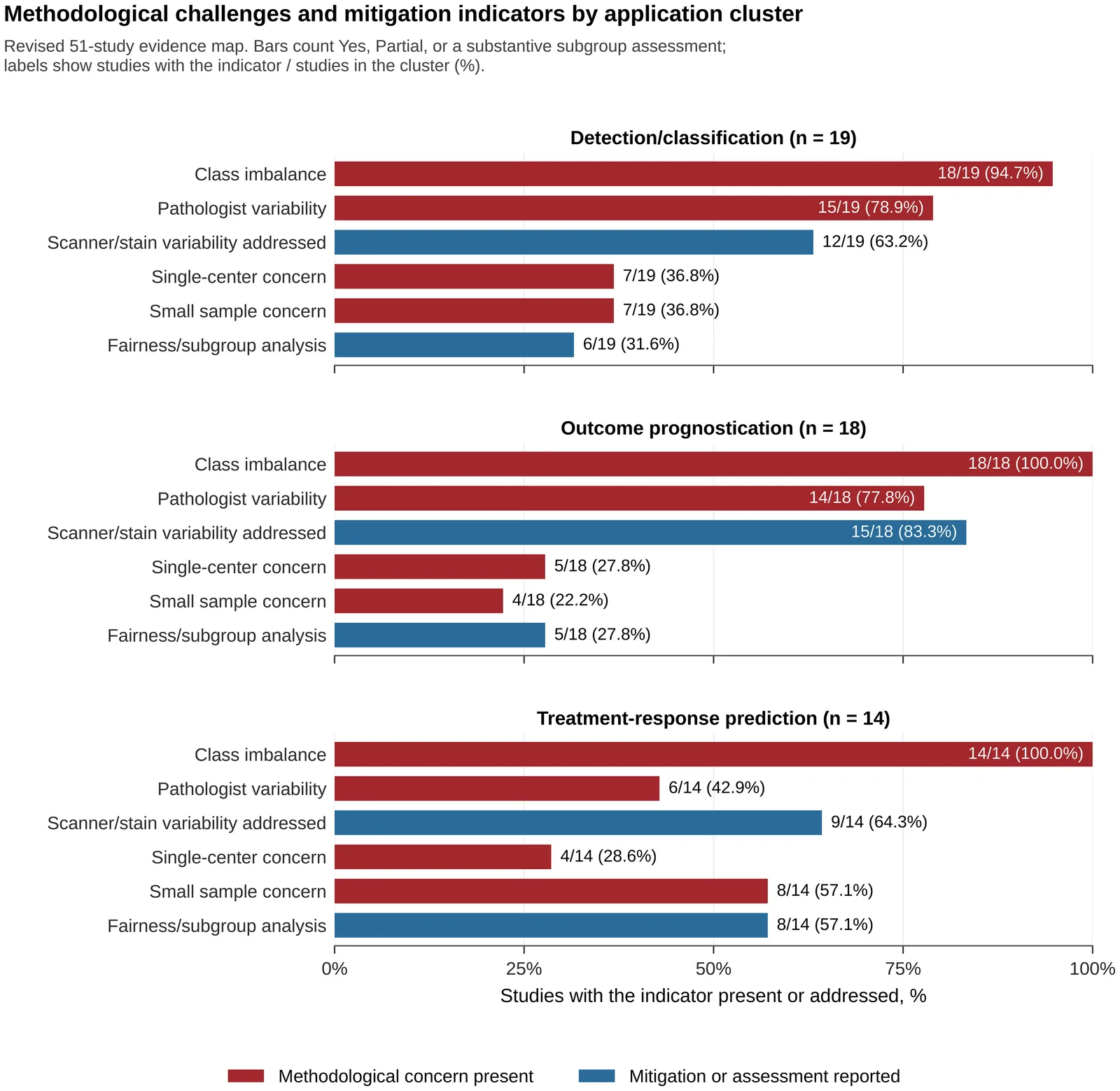
Methodological challenges by application cluster.

Technical robustness was addressed more frequently than in the previous analysis. Scanner or stain variability was assessed or mitigated in 63.2% of detection/classification studies (12/19), 83.3% of outcome prognostication studies (15/18), and 64.3% of treatment-response prediction studies (9/14). Fairness or substantive subgroup analysis remained less consistently reported, occurring in 31.6% of detection/classification studies (6/19), 27.8% of prognostication studies (5/18), and 57.1% of treatment-response prediction studies (8/14). Thus, although treatment-response studies showed comparatively greater attention to subgroup performance, fairness assessment was still absent from a substantial proportion of the literature.

Single-center and small-sample concerns also persisted. Single-center limitations were identified in 36.8% of detection/classification studies (7/19), 27.8% of outcome prognostication studies (5/18), and 28.6% of treatment-response prediction studies (4/14). Small-sample concerns were present in 36.8% of detection/classification studies (7/19), 22.2% of prognostication studies (4/18), and 57.1% of treatment-response prediction studies (8/14). Overall, the figure indicates that TIL-ML research remains affected by pervasive class imbalance and annotation variability, while technical robustness, fairness assessment, and study-design limitations are addressed inconsistently across application clusters. Treatment-response prediction studies were particularly affected by small samples, whereas prognostication studies more frequently evaluated scanner or stain-related robustness.

Overall, reporting completeness across studies was moderate to high, with the majority of TRIPOD+AI items reported in more than 70% of studies (**Figure 7a**). Items related to study objectives, data sources, model development, and performance reporting were consistently well described, with near-complete adherence across studies. In particular, items concerning data description (Items 4–5), model specification (Items 9–11), and performance evaluation (Items 12a–12e) demonstrated high reporting rates. However, several key deficiencies were identified. Items related to outcome definition (Item 6c), model interpretation (Item 23b), and clinical applicability and implementation (Items 24–26) were less frequently reported. Notably, clinical interpretation of model performance (Item 23b) was often absent, with many studies reporting technical metrics such as AUC or F1-score without contextualizing their clinical relevance. Similarly, reporting of model limitations (Item 24), although present in most studies, varied in depth and often lacked discussion of generalizability and real-world implementation constraints. Items related to reproducibility and transparency (Items 27a–27c) showed variable reporting.

**Figure 7:**
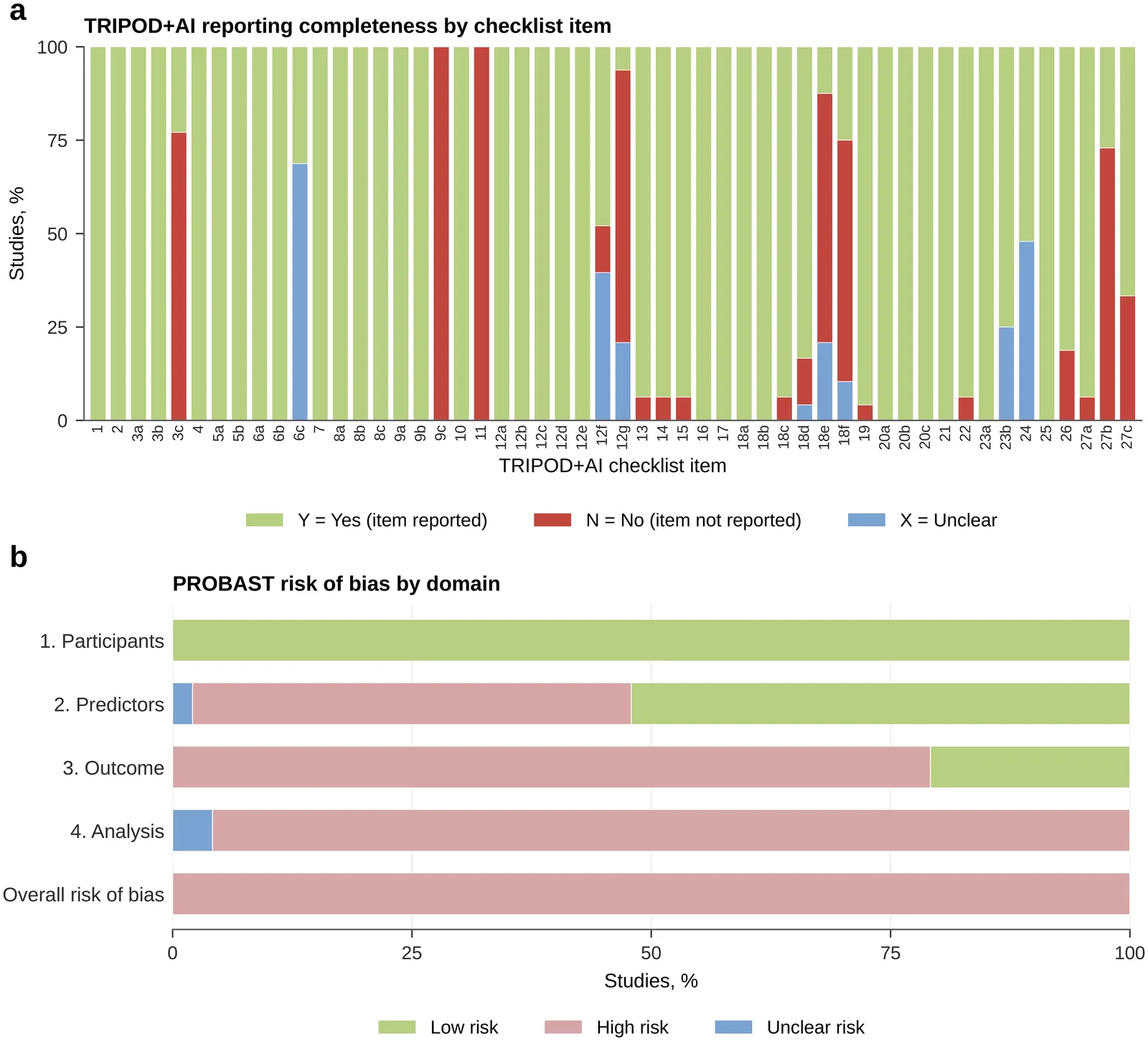
Methodological quality of the included studies: **(a)** reporting completeness by TRIPOD+AI checklist item; **(b)** PROBAST risk of bias by domain.

The PROBAST assessment revealed substantial methodological limitations across the included studies (**Figure 7b**). Overall, all studies were classified as having a high risk of bias, driven primarily by concerns within the analysis and outcome domains. The participants domain demonstrated low risk of bias across all studies, reflecting generally adequate reporting of data sources and sample characteristics. Similarly, the predictors domain showed predominantly low risk, indicating that input variables were typically well defined and consistently measured. In contrast, the outcome domain exhibited a high proportion of studies at high risk of bias. This was largely attributable to variability in outcome definitions, reliance on surrogate or weakly standardized labels, and limited reporting of annotation procedures and interobserver variability. The analysis domain represented the most critical source of bias, with the majority of studies classified as high risk. Common issues included small sample sizes relative to model complexity, lack of external validation, potential overfitting due to internal optimization, and absence of calibration or clinical utility analyses. Although some studies employed cross-validation or held-out test sets, these approaches were often insufficient to mitigate concerns regarding model generalizability. The overall risk of bias was therefore judged as high across all included studies, reflecting consistent methodological limitations that may affect the reliability and clinical applicability of reported model performance.

## 4 Discussion

This systematic review and evidence-gap analysis demonstrates that ML-based analysis of TILs in breast cancer is not a single methodological problem, but a set of distinct computational and translational tasks. Across 51 primary histopathology-image machine learning studies, we assessed three application clusters: TIL detection/classification, outcome prognostication, and treatment-response prediction. The main finding is that these clusters differ not only in clinical purpose but also in data structure, annotation requirements, model families, validation practices, and readiness for clinical translation. Therefore, future TIL-ML studies should not be planned using a generic “automated TIL scoring” framework; instead, study design should be aligned with the intended application.

The clinical motivation for automated TIL assessment is strong. The International TIL Working Group has provided standardized recommendations for visual stromal TIL assessment in breast cancer, emphasizing the stromal compartment and the need for reproducible scoring (9). Ring studies have shown that standardized TIL evaluation is feasible but still vulnerable to interobserver variability, particularly when scoring is performed across pathologists with different experience levels or across heterogeneous tissue regions (76). More recent reviews from the International Immuno-Oncology Biomarker Working Group have further emphasized that TILs have prognostic and predictive relevance, especially in triple-negative and HER2-positive breast cancer, and that they may become increasingly important in the era of immune-checkpoint inhibition (4, 77). Against this background, automated TIL assessment is attractive because it could improve reproducibility, quantify spatial immune patterns, and generate patient-level biomarkers from routine H&E or IHC slides. However, our findings suggest that the computational literature has not yet reached the level of validation and clinical-utility evidence needed for routine implementation.

Our results extend prior reviews of automated TIL scoring in breast cancer. The most directly comparable systematic review summarized automated scoring methods and reported that segmentation, object detection, and convolutional neural networks were the dominant approaches (78). That review was valuable for describing the technical landscape, but the present study shifts the emphasis from model performance alone to application-specific translational readiness. By separating the evidence into detection/classification, prognostication, and treatment-response prediction clusters, we show that the apparent maturity of the field depends strongly on the target task. Detection/classification studies were largely technical proof-of-concept studies, whereas prognostication and treatment-response prediction studies were closer to patient-level decision-making but still lacked several requirements for clinical translation.

A clear translational gradient was observed. External validation was reported in 32% of detection/classification studies, compared with 61% of outcome prognostication studies and 57% of treatment-response prediction studies. Clinical comparator reporting followed a similar pattern, increasing from 63% in detection/classification studies to 94% in the prognostication cluster and 93% in the treatment-response cluster. This suggests that studies closer to clinical decision-making more often evaluate whether machine learning-derived TIL features add information beyond pathologist assessment or established clinicopathological variables. However, these findings also show that greater clinical proximity does not necessarily translate into clinical readiness. Outcome calibration was reported in only one outcome-prognostication study (1/18, 5.6%) and in none of the applicable detection/classification (0/19) or treatment-response prediction studies (0/14). Decision-curve analysis or net-benefit assessment was similarly reported in only one outcome-prognostication study (1/18, 5.6%) and in none of the applicable detection/classification (0/19) or treatment-response prediction studies (0/14). These findings indicate that most models remain insufficiently evaluated for calibrated risk estimation and threshold-based clinical decision-making (79).

The near absence of calibration is particularly important for prognostic and treatment-response prediction studies. A model that ranks patients well may still systematically overestimate or underestimate individual risk, which can mislead treatment escalation or de-escalation decisions. Calibration plots, calibration intercepts and slopes, Brier scores, and recalibration procedures are standard components of modern clinical prediction-model evaluation (79). Similarly, decision-curve analysis is needed when the intended use is clinical decision support, because it estimates whether model-guided decisions provide net benefit across threshold probabilities (80). In this review, the near absence of decision-curve analysis across all clusters indicates that most TIL-ML models remain in the analytical or exploratory phase rather than the implementation phase.

The clusters also differed in their methodological constraints, and each shows a distinct strength/weakness trade-off. Detection/classification studies showed the greatest architectural diversity of any cluster but the lowest rate of external validation; they were predominantly technical in orientation and strongly shaped by annotation granularity. These studies often required pixel-level, nucleus-level, patch-level, or region-level ground truth, explaining the frequent use of segmentation, object-detection, and cell-classification pipelines. External validation remained limited, being reported in 32% of studies, although clinical or pathologist comparators were reported in 63%. Scanner or stain variability was addressed in 63.2%, while single-center and small-sample concerns were each identified in 36.8% of studies. This matters mechanistically: H&E stain intensity and hue vary with reagent batch, protocol, and scanner, directly perturbing the color- and texture-based features many TIL-detection pipelines use to separate lymphocyte nuclei from stromal or tumor cells (81). A model trained under one staining protocol can therefore under- or over-call TILs when applied to slides stained or scanned differently unless stain normalization, color augmentation, or multi-site training data are used. This means that reporting a robustness check does not by itself establish robustness. For this cluster, the central challenge is therefore not simply improving Dice, F1, or AUC values, but demonstrating that technical performance remains stable across institutions, scanners, staining protocols, tissue-preparation workflows, and annotation practices (**Table 2**).

**Table 2:** Planning checklist for TIL detection/classification studies.

Domain	Recommendation	Rationale and supporting standard
Intended use	Define whether the model detects lymphocytes, classifies TILs versus non-TILs, segments tissue compartments, estimates sTIL percentage, or supports pathologist review.	Avoids mixing technical detection with clinical prediction; aligns with CLAIM and MI-CLAIM reporting principles (90, 91).
Annotation	Report annotation level, number of annotators, annotator expertise, consensus procedure, and interobserver agreement.	Pathologist variability was present in all detection/classification studies; TIL-WG and CLAIM emphasize transparent ground-truth definition (9, 76, 90).
TIL compartment	Distinguish stromal TILs, intratumoral TILs, hot-spot regions, and immune-rich areas where applicable.	TIL-WG recommends stromal TIL reporting, while computational models often mix compartments (9).
Ground truth	Use shared datasets or contribute annotations to public benchmarks where possible; clearly distinguish private, public, and challenge datasets.	Bespoke ground truth limits comparability; benchmark-based evaluation improves reproducibility (90, 92).
Unit of analysis	Pre-specify patch-, cell-, region-, slide-, and patient-level outputs; avoid presenting patch-level metrics as patient-level evidence.	Unit-of-analysis mismatch was a recurring limitation.
Performance	Report task-appropriate metrics, including Dice, F1, precision, recall, ROC-AUC, PR-AUC, and bootstrap 95% confidence intervals.	Class imbalance was universal; PR-AUC and uncertainty estimates are important for imbalanced tasks (23, 90).
Scanner/stain robustness	Report scanner, magnification, staining protocol, tissue preparation, stain normalization, and cross-scanner or cross-site sensitivity analyses.	Scanner/stain variability was rarely addressed in the evidence map.
External validation	Validate on at least one independent non-overlapping cohort, ideally from a different laboratory, scanner, or institution.	External validation was reported in 31.6% of detection/classification studies.
Clinical comparator	Benchmark outputs against pathologist visual TIL assessment or consensus scores when the intended use is TIL scoring.	Clinical comparator reporting was limited in this cluster.
Reproducibility	Release code, model weights, inference pipeline, annotation schema, and example data where legally possible.	Code and data availability were incomplete across all clusters (90, 91).

AUC, area under the curve; CI, confidence interval; CLAIM, Checklist for Artificial Intelligence in Medical Imaging; F1, harmonic mean of precision and recall; MI-CLAIM, Minimum Information about Clinical Artificial Intelligence Modeling; PR-AUC, area under the precision–recall curve; ROC-AUC, area under the receiver operating characteristic curve; sTILs, stromal tumor-infiltrating lymphocytes; TILs, tumor-infiltrating lymphocytes; TIL-WG, International Immuno-Oncology Biomarker Working Group on Breast Cancer/Tumor-Infiltrating Lymphocytes Working Group.

Outcome prognostication studies were more clinically anchored but methodologically heterogeneous. These studies more often used patient-level outcomes and clinical comparators, but they differed substantially in endpoint definition, follow-up duration, breast cancer subtype, treatment background, and feature construction. Survival endpoints included overall survival, disease-free survival, recurrence-free survival, invasive disease-free survival, progression-free interval, and disease-specific survival. Such heterogeneity makes it difficult to compare results across studies and reinforces the need for standardized endpoint reporting, consistent follow-up description, and transparent separation of prognostic from predictive claims. Reporting should align with tumor-marker prognostic standards and breast cancer endpoint frameworks, including REMARK and STEEP 2.0 (82, 83) (**Table 3**).

**Table 3:** Planning checklist for TIL-based outcome prognostication studies.

Domain	Recommendation	Rationale and supporting standard
Clinical question	State whether the model is prognostic, predictive, or both; avoid implying treatment prediction from observational prognostic association.	Prognostic and predictive claims were often difficult to separate; TRIPOD+AI and REMARK require clear objectives (23, 83).
Endpoint	Pre-specify one primary survival endpoint and define secondary endpoints using standardized breast cancer terminology.	Endpoint heterogeneity limits comparability; STEEP 2.0 provides standardized definitions (82).
Follow-up	Report median follow-up, number of events, censoring, and events per candidate predictor or model feature.	Required for interpreting survival models and risk of overfitting (23, 83).
TIL feature definition	Define whether features represent sTILs, iTILs, CD8/CD20 immune cells, immune density, spatial clustering, tumor-immune proximity, or composite TME scores.	Computational TIL features were heterogeneous and not always biologically equivalent.
Treatment context	Report adjuvant and neoadjuvant treatments; stratify or adjust for treatment when feasible.	Treatment heterogeneity can confound prognostic associations.
Model development	Describe feature selection, cross-validation, hyperparameter tuning, and leakage prevention at patient level.	PROBAST+AI emphasizes analysis-domain risk of bias (95).
Clinical comparator	Compare against clinicopathological models, pathologist TIL scores, grade, stage, nodal status, molecular subtype, and established biomarkers.	Comparator reporting was common but should be standardized.
Incremental value	Report change in C-index, likelihood ratio, calibration, net reclassification, or other prespecified incremental-value metrics over clinical baseline models.	Incremental value was limited, especially outside response-prediction studies (23).
Calibration and utility	Report calibration plots, calibration slope/intercept, Brier score, and decision-curve analysis if the model is intended for risk stratification.	Calibration and DCA were major gaps in the evidence map (79, 80).
External validation	Validate in a geographically or temporally independent cohort with non-overlapping slides/patients.	External validation was present in only 61.1% of prognostication studies.
Fairness/subgroups	Evaluate performance across subtype, age, ethnicity/geography, tumor grade, treatment group, and scanner/stain source where possible.	Fairness/subgroup analysis was rare.

AI, artificial intelligence; CD8, cluster of differentiation 8; CD20, cluster of differentiation 20; C-index, concordance index; DCA, decision-curve analysis; iTILs, intratumoral tumor-infiltrating lymphocytes; NRI, net reclassification improvement; PROBAST+AI, Prediction Model Risk Of Bias Assessment Tool–Artificial Intelligence; REMARK, Reporting Recommendations for Tumor Marker Prognostic Studies; STEEP 2.0, Standardized Definitions for Efficacy End Points in Adjuvant Breast Cancer Clinical Trials, version 2.0; sTILs, stromal tumor-infiltrating lymphocytes; TILs, tumor-infiltrating lymphocytes; TME, tumor microenvironment; TRIPOD+AI, Transparent Reporting of a Multivariable Prediction Model for Individual Prognosis or Diagnosis–Artificial Intelligence.

Treatment-response prediction studies were the most clinically oriented cluster by comparator use and incremental-value reporting, but this clinical proximity was not matched by calibration or decision-analytic evidence: clinical comparator reporting reached 93% of studies and the highest frequency of incremental-value analysis (64%). External validation was reported in 57%, which was substantially higher than in detection/classification studies but slightly lower than in outcome prognostication studies (61%). Nevertheless, outcome calibration and decision-curve analysis were absent from this cluster, indicating that frequent clinical benchmarking did not translate into evidence of calibrated prediction or decision-level benefit. Among the primary evidence-map studies, we did not identify a mature body of ML-based TIL models predicting response to immune-checkpoint inhibition. This is an important gap because TILs are biologically and clinically relevant to anti-tumor immunity and may be especially informative in triple-negative breast cancer treated with immunotherapy (4, 77, 84, 85). This gap likely reflects several practical barriers: immunotherapy trials in breast cancer remain smaller and more heterogeneous in regimen than neoadjuvant chemotherapy trials; immune-related response patterns, including pseudo-progression and delayed response, complicate the definition of a stable ground-truth label for supervised learning (86); and no large, annotated, multi-institutional immunotherapy-response TIL cohort comparable to established chemotherapy-focused cohorts currently exists. Future response-prediction studies should therefore move beyond chemotherapy-only settings and evaluate whether computational TIL features improve prediction of immunotherapy response, treatment de-escalation, or escalation decisions (**Table 4**).

**Table 4:** Planning checklist for TIL-based treatment-response prediction studies.

Domain	Recommendation	Rationale and supporting standard
Treatment setting	Define therapy type, regimen, treatment line, and whether the model targets chemotherapy, anti-HER2 therapy, immunotherapy, or combined regimens.	The current evidence was dominated by neoadjuvant chemotherapy.
Response endpoint	Use standardized pCR definitions, preferably ypT0/is ypN0 or another explicitly stated CTNeoBC/FDA-compatible definition; report residual cancer burden where available.	pCR definitions varied; CTNeoBC and FDA guidance support standardized endpoint reporting (93).
Specimen timing	Specify whether tissue was pretreatment biopsy, surgical resection, post-treatment tissue, or paired tissue.	Treatment-response models should use pretreatment material if intended for pretherapy decision support.
Sampling adequacy	Report tumor area, biopsy adequacy, minimum tissue threshold, number of cores, and exclusion criteria for insufficient tissue.	Biopsy-only sampling bias is a cluster-specific concern.
TIL and TME features	Define whether the model uses TIL density, sTILs, iTILs, immune-cell subtype, spatial TIL pattern, tumor/stroma features, or multimodal biomarkers.	Multimodal integration was common but inconsistently reported.
Model performance	Report patient-level AUC, sensitivity, specificity, calibration, confidence intervals, and decision thresholds.	Patient-level prediction is the relevant unit for treatment decisions (23).
Clinical comparator	Compare against clinical subtype, grade, stage, ER/PR/HER2 status, Ki-67, PD-L1, pathologist TIL score, and standard clinical models.	Comparator reporting was common but not uniform.
Incremental value	Test whether ML-derived TIL/TME features improve prediction beyond clinical + pathology + IHC baselines.	Incremental-value analysis was strongest in this cluster but still incomplete.
Calibration and DCA	Report calibration and decision-curve analysis at clinically meaningful treatment thresholds.	No primary studies reported DCA, and calibration was absent in this cluster (79, 80).
External validation	Validate on independent, non-overlapping cohorts, preferably across institutions, countries, scanners, and treatment regimens.	External validation was present in 57.1% but independence was not always verifiable.
Immunotherapy gap	Include immune-checkpoint inhibitor response endpoints where relevant, particularly in TNBC.	No mature AI-based TIL immunotherapy-response evidence was identified in the primary evidence map (4, 77).
Reproducibility	Release code, feature definitions, model weights, and trained pipelines where possible.	Reproducibility remains limited across clusters (90, 91).

AI, artificial intelligence; AUC, area under the curve; CI, confidence interval; CTNeoBC, Collaborative Trials in Neoadjuvant Breast Cancer; DCA, decision-curve analysis; ER, estrogen receptor; FDA, US Food and Drug Administration; HER2, human epidermal growth factor receptor 2; IHC, immunohistochemistry; iTILs, intratumoral tumor-infiltrating lymphocytes; Ki-67, marker of proliferation Ki-67; ML, machine learning; pCR, pathological complete response; PD-L1, programmed death-ligand 1; PR, progesterone receptor; RCB, residual cancer burden; sTILs, stromal tumor-infiltrating lymphocytes; TILs, tumor-infiltrating lymphocytes; TME, tumor microenvironment; TNBC, triple-negative breast cancer; ypN0, no residual nodal disease after neoadjuvant therapy; ypT0/is, no residual invasive tumor in the breast, with or without residual *in situ* disease, after neoadjuvant therapy.

Another important finding is that methodological challenges were not isolated problems but structural features of the evidence base. Class imbalance was present across all three clusters. Pathologist variability was common in detection/classification (78.9%) and outcome prognostication studies (77.8%) but less frequently identified in treatment-response prediction studies (42.9%), probably because the latter more often used patient-level treatment outcomes rather than pathologist-assigned TIL labels as the primary endpoint. Nevertheless, annotation-related variability may still affect treatment-response models that depend on pathologist-informed tissue compartments or TIL features. Scanner or stain variability was assessed or mitigated in 63.2% of detection/classification studies, 83.3% of outcome prognostication studies, and 64.3% of treatment-response prediction studies. These findings indicate growing recognition of technical domain shift, although the depth and quality of the reported assessments varied and cannot be inferred from their presence alone. Fairness or substantive subgroup analysis was reported in 31.6%, 27.8%, and 57.1% of the three clusters, respectively. Thus, fairness assessment was not absent, but remained incomplete, particularly in detection/classification and prognostication studies.

Model-family distribution further demonstrated that methodological choices were task-dependent. Detection/classification studies showed the greatest architectural diversity, with segmentation or object-detection and classical or handcrafted machine learning each used in five studies, followed by CNN/deep-learning approaches in four studies and several less frequent model families. Outcome prognostication was concentrated around classical or handcrafted machine learning (six studies), segmentation or object-detection approaches (five studies), and CNN/deep learning (four studies), with only limited use of hybrid and foundation or transformer-based architectures. Treatment-response prediction showed the most concentrated distribution, comprising classical or handcrafted machine learning in seven studies, CNN/deep learning in five, and segmentation or object detection in two. This task-dependent heterogeneity, together with differences in endpoints, input representations, and validation procedures, supports narrative rather than pooled quantitative synthesis.

Weakly supervised, self-supervised, and foundation or transformer-based approaches represented only a small proportion of the included evidence and were absent from the treatment-response prediction cluster. Multiple-instance learning was uncommon, despite its suitability for whole-slide image analysis with weak slide-level labels. Similarly, transformer and foundation-model approaches appeared rarely in this TIL-specific literature, even though recent computational pathology research has rapidly advanced toward large-scale self-supervised, whole-slide, and vision-language foundation models (87–89). This suggests that TIL-ML research has not fully incorporated the broader methodological shift in computational pathology. Nevertheless, foundation models should not be viewed as a shortcut to clinical readiness. They may reduce annotation burden and improve feature transfer, but they still require task-specific validation, calibration, interpretability, subgroup assessment, and external evaluation before being used for clinical decision support.

The evidence base was also characterized by repeated reuse of a relatively small number of named public and challenge cohorts. These cohorts differ in modality and scale — TCGA-BRCA and METABRIC are large, multi-institutional H&E/molecular resources, whereas TIGER and BCSS are smaller, purpose-built segmentation/detection challenge sets — and a dedicated review of TIL-specific public datasets is available for readers requiring full characteristics (13). Reuse of established cohorts facilitates benchmarking, method comparison, and secondary validation, but repeated analyses of the same underlying patient populations do not constitute independent patient-level evidence. Consequently, the number of published studies may overstate the diversity of represented populations, institutions, and clinical contexts. Future studies should clearly identify cohort overlap and prioritize genuinely independent, geographically diverse, and prospectively collected external-validation datasets.

Reproducibility remained incomplete across all application clusters. Code was available for 47% of detection/classification studies, 39% of outcome prognostication studies, and 43% of treatment-response prediction studies. Model weights were less frequently available, being reported in 33% of applicable detection/classification studies, none of the applicable prognostication studies, and 7% of treatment-response prediction studies. Data availability was recorded in 63%, 50%, and 29%, respectively. However, because studies using named public datasets were classified as having available data, these percentages primarily indicate accessibility of the underlying source cohort and should not be interpreted as evidence that the exact analytic dataset, preprocessing workflow, train–test partition, annotations, or derived features were fully reproducible. Clinical translation requires availability of these study-specific components in addition to access to the original public data.

Taken together, the findings support the use of cluster-specific planning checklists rather than a single generic reporting recommendation. Existing standards already provide many of the required elements. TRIPOD+AI provides guidance for prediction-model reporting; CLAIM and MI-CLAIM support transparent reporting of AI in medical imaging and clinical AI modeling; STARD-AI is relevant for AI diagnostic accuracy studies; DECIDE-AI addresses early clinical evaluation of AI decision-support systems; PROBAST+AI supports risk-of-bias and applicability assessment for prediction models; REMARK and STEEP 2.0 are relevant to prognostic biomarker and breast cancer endpoint reporting; and CTNeoBC/FDA frameworks support standardized pCR reporting in neoadjuvant studies (23, 24, 82, 83, 90–95). The contribution of the present review is to translate these general standards into TIL-ML application-specific planning checklists derived from the evidence-gap analysis (**Tables 2**–**4**).

This review has limitations. First, the main evidence map was restricted to 51 primary histopathology-image ML studies. This decision improved internal consistency but means that cross-modal radiomics, transcriptomic, and molecular TIL-related studies were not included in the primary results. Second, the included studies often addressed more than one task, and assigning each study to a single dominant application cluster may have simplified overlapping aims. Third, extraction was limited by incomplete reporting in the original articles, particularly for sample size, slide counts, scanner details, stain protocols, code availability, data availability, and validation independence. Computational cost (runtime, hardware requirements, model size) was reported too inconsistently across the 51 studies to be extracted systematically and is itself an evidence-gap finding rather than an omission of this review. Fourth, because the included studies used non-comparable metrics and endpoints, quantitative meta-analysis was not appropriate. Finally, the evidence-gap categories were descriptive and intended for research planning rather than formal statistical inference.

Several emerging directions could help close the gaps identified above. Federated learning offers a route to the multicenter training data our results show is currently lacking (only 6/19 detection/classification studies used multicenter data) without requiring raw slide transfer across institutions (96). Domain adaptation methods are a direct response to the scanner/stain-robustness gaps documented in this review (81). Model-uncertainty quantification would complement the near-total absence of calibration reporting we identified (97). Fairness and subgroup evaluation, reported in only 27.8–57.1% of studies across clusters, should be treated as a standard reporting element rather than an optional analysis. None of the 51 included studies addressed regulatory approval pathways or clinical-workflow integration burden, underscoring that deployment-readiness, not further model development, is the field’s principal unmet need.

## 5 Conclusion

In conclusion, TIL-ML research in breast cancer is technically active and increasingly clinically oriented but remains incompletely validated for routine implementation. Detection/classification studies showed the greatest architectural diversity but lower external-validation rates than prognostication and treatment-response studies. Clinically oriented studies frequently incorporated clinical or pathologist comparators, and treatment-response studies more often assessed incremental value; however, outcome calibration and decision-curve analysis were almost entirely absent. Code, model-weight, and study-specific data sharing remained incomplete, while repeated reuse of a small number of public cohorts limited the independence and population diversity of the evidence base. Scanner- and stain-related robustness was addressed in a majority of studies, but fairness, subgroup performance, sample-size adequacy, and genuinely independent external validation were evaluated inconsistently. The next phase of the field should prioritize calibrated, clinically benchmarked, externally validated, reproducible, and decision-relevant models over further single-center model development. The proposed cluster-specific planning checklists are intended to support this transition from exploratory computational pathology toward clinically translatable TIL-ML evidence.

## Data Availability

The original contributions presented in the study are included in the article/**Supplementary Material**. Further inquiries can be directed to the corresponding author.

## References

[1] World Health Organization . Breast cancer key facts, in: Breast Cancer (2024). Available online at: https://www.who.int/news-room/fact-sheets/detail/breast-cancer (Accessed August 1, 2024).

[2] Zhang S Jin Z Bao L Shu P . The global burden of breast cancer in women from 1990 to 2030: assessment and projection based on the global burden of disease study 2019. Front Oncol. (2024) 14. doi: 10.3389/fonc.2024.1364397 38966067 PMC11222408

[3] Karibayeva I Turdaliyeva B Zainal NZ Bagiyarova F . The prevalence of depression symptoms among advanced breast cancer patients: A systematic review and meta-analysis. Arch Balkan Med Union. (2020) 55:651–60. doi: 10.31688/ABMU.2020.55.4.12

[4] El Bairi K Haynes HR Blackley E Fineberg S Shear J Turner S . The tale of TILs in breast cancer: a report from the international immuno-oncology biomarker working group. NPJ Breast Cancer. (2021) 7:150–67. doi: 10.1038/s41523-021-00346-1 34853355 PMC8636568

[5] Yu X Zhang Z Wang Z Wu P Qiu F Huang J . Prognostic and predictive value of tumor-infiltrating lymphocytes in breast cancer: a systematic review and meta-analysis. Clin Transl Oncol. (2016) 18:497–506. doi: 10.1007/s12094-015-1391-y 26459255 PMC4823351

[6] Muenst S Schaerli AR Gao F Däster S Trella E Droeser RA . Expression of programmed death ligand 1 (PD-L1) is associated with poor prognosis in human breast cancer. Breast Cancer Res Treat. (2014) 146:15–24. doi: 10.1007/s10549-014-2988-5 24842267 PMC4180714

[7] Ciarka A Piątek M Pęksa R Kunc M Senkus E . Tumor-infiltrating lymphocytes (TILs) in breast cancer: Prognostic and predictive significance across molecular subtypes. Biomedicines. (2024) 12:763–79. doi: 10.3390/biomedicines12040763 38672117 PMC11048219

[8] Tajbakhsh N Jeyaseelan L Li Q Chiang JN Wu Z Ding X . Embracing imperfect datasets: A review of deep learning solutions for medical image segmentation. Med Image Anal. (2020) 63:101693. doi: 10.1016/j.media.2020.101693 32289663

[9] Salgado R Denkert C Demaria S Sirtaine N Klauschen F Pruneri G . The evaluation of tumor-infiltrating lymphocytes (TILs) in breast cancer: recommendations by an International TILs Working Group 2014. Ann Oncol. (2015) 26:259–71. doi: 10.1093/annonc/mdu450 25214542 PMC6267863

[10] Laenkholm A-V Callagy G Balancin M Bartlett JMS Sotiriou C Marchio C . Incorporation of TILs in daily breast cancer care: how much evidence can we bear? Virchows Arch. (2022) 480:147–62. doi: 10.1007/s00428-022-03276-w 35043236

[11] Zgura A Galesa L Bratila E Anghel R . Relationship between tumor infiltrating lymphocytes and progression in breast cancer. Maedica (Bucur). (2018) 13:317–20. doi: 10.26574/maedica.2018.13.4.317 30774731 PMC6362880

[12] Sarker IH . Deep learning: A comprehensive overview on techniques, taxonomy, applications and research directions. SN Comput Sci. (2021) 2:420–40. doi: 10.1007/S42979-021-00815-1 34426802 PMC8372231

[13] Fiorin A López Pablo C Lejeune M Hamza Siraj A Della Mea V . Enhancing AI research for breast cancer: A comprehensive review of tumor-infiltrating lymphocyte datasets. J Imaging Inf Med. (2024) 37:1–13. doi: 10.1007/s10278-024-01043-8 38806950 PMC11612116

[14] Thagaard J Broeckx G Page DB Jahangir CA Verbandt S Kos Z . Pitfalls in machine learning‐based assessment of tumor‐infiltrating lymphocytes in breast cancer: A report of the International Immuno‐Oncology Biomarker Working Group on Breast Cancer. J Pathol. (2023) 260:498–513. doi: 10.1002/path.6155 37608772 PMC10518802

[15] Miao R Toth R Zhou Y Madabhushi A Janowczyk A . Quick Annotator: an open‐source digital pathology based rapid image annotation tool. J Pathol Clin Res. (2021) 7:542–7. doi: 10.1002/cjp2.229 34288586 PMC8503896

[16] Ochi T Bianchini G Ando M Nozaki F Kobayashi D Criscitiello C . Predictive and prognostic value of stromal tumour-infiltrating lymphocytes before and after neoadjuvant therapy in triple negative and HER2-positive breast cancer. Eur J Cancer. (2019) 118:41–8. doi: 10.1016/j.ejca.2019.05.014 31302586

[17] Page MJ McKenzie JE Bossuyt PM Boutron I Hoffmann TC Mulrow CD . The PRISMA 2020 statement: an updated guideline for reporting systematic reviews. Bmj. (2021) 372:1–9. doi: 10.1136/bmj.n71 33782057 PMC8005924

[18] Jia Y Wu R Lu X Duan Y Zhu Y Ma Y . Deep learning with transformer or convolutional neural network in the assessment of tumor-infiltrating lymphocytes (TILs) in breast cancer based on US images: a dual-center retrospective study. Cancers (Basel). (2023) 15:838–42. doi: 10.3390/cancers15030838 36765796 PMC9913836

[19] Liu B Gu X Xie D Zhao B Han D Zhang Y . An ultrasound-based machine learning model for predicting tumor-infiltrating lymphocytes in breast cancer. Technol Cancer Res Treat. (2025) 24. doi: 10.1177/15330338251334453 40241518 PMC12035158

[20] Wu X Li C . Development of a prognostic model for breast cancer patients based on intratumoral tumor-infiltrating lymphocytes using machine learning algorithms. Discover Oncol. (2025) 16:762. doi: 10.1007/S12672-025-02585-1 40366513 PMC12078914

[21] Banerjee S Tiwari VK Raman K Inayatullah M . Tumor-infiltrating lymphocytes display prognostic signatures associated with chemotherapy response in TNBC patients. iScience. (2026) 29:116295. doi: 10.1016/J.ISCI.2026.116295 42291245 PMC13264094

[22] Collins GS Dhiman P Andaur Navarro CL Ma J Hooft L Reitsma JB . Protocol for development of a reporting guideline (TRIPOD-AI) and risk of bias tool (PROBAST-AI) for diagnostic and prognostic prediction model studies based on artificial intelligence. BMJ Open. (2021) 11:e048008. doi: 10.1136/BMJOPEN-2020-048008 34244270 PMC8273461

[23] Collins GS Moons KGM Dhiman P Riley RD Beam AL Van Calster B . TRIPOD+AI statement: updated guidance for reporting clinical prediction models that use regression or machine learning methods. BMJ. (2024) 385. doi: 10.1136/BMJ-2023-078378 38626948 PMC11019967

[24] Moons KGM Wolff RF Riley RD Whiting PF Westwood M Collins GS . PROBAST: A tool to assess risk of bias and applicability of prediction model studies: Explanation and elaboration. Ann Intern Med. (2019) 170:W1–W33. doi: 10.7326/M18-1377 30596876

[25] Basavanhally AN Ganesan S Agner S Monaco JP Feldman MD Tomaszewski JE . Computerized image-based detection and grading of lymphocytic infiltration in HER2+ breast cancer histopathology. IEEE Trans BioMed Eng. (2010) 57:642–53. doi: 10.1109/TBME.2009.2035305 19884074

[26] Turkki R Linder N Kovanen PE Pellinen T Lundin J . Antibody-supervised deep learning for quantification of tumor-infiltrating immune cells in hematoxylin and eosin stained breast cancer samples. J Pathol Inform. (2016) 7:38. doi: 10.4103/2153-3539.189703 27688929 PMC5027738

[27] Amgad M Sarkar A Srinivas C Redman R Ratra S Bechert CJ . Joint region and nucleus segmentation for characterization of tumor infiltrating lymphocytes in breast cancer. In: Medical Imaging 2019: Digital Pathology. Bellingham, WA: SPIE (2019). p. 129–36. doi: 10.1117/12.2512892 PMC6988758 31997849

[28] Le H Gupta R Hou L Abousamra S Fassler D Torre-Healy L . Utilizing automated breast cancer detection to identify spatial distributions of tumor-infiltrating lymphocytes in invasive breast cancer. Am J Pathol. (2020) 190:1491–504. doi: 10.1016/j.ajpath.2020.03.012 32277893 PMC7369575

[29] Lee S Amgad M Mobadersany P McCormick M Pollack BP Elfandy H . Interactive classification of whole-slide imaging data for cancer researchers. Cancer Res. (2021) 81:1171–7. doi: 10.1158/0008-5472.CAN-20-0668 33355190 PMC8026494

[30] Narayanan PL Raza SEA Hall AH Marks JR King L West RB . Unmasking the immune microecology of ductal carcinoma in situ with deep learning. NPJ Breast Cancer. (2021) 7:19–33. doi: 10.1038/s41523-020-00205-5 33649333 PMC7921670

[31] Negahbani F Sabzi R Pakniyat Jahromi B Firouzabadi D Movahedi F Kohandel Shirazi M . PathoNet introduced as a deep neural network backend for evaluation of Ki-67 and tumor-infiltrating lymphocytes in breast cancer. Sci Rep. (2021) 11:8489–502. doi: 10.1038/s41598-021-86912-w 33875676 PMC8055887

[32] Schirris Y Engelaer M Panteli A Horlings HM Gavves E Teuwen J . WeakSTIL: Weak whole-slide image level stromal tumor infiltrating lymphocyte scores are all you need. In: Medical Imaging 2022: Digital and Computational Pathology. Bellingham, WA: SPIE (2022). p. 55–9. doi: 10.1117/12.2611528

[33] Shephard AJ Jahanifar M Wang R Dawood M Graham S Sidlauskas K . An automated pipeline for tumour-infiltrating lymphocyte scoring in breast cancer. In: Arxiv Preprint Arxiv:231106185. Ithaca, NY: Cornell University Library, arXiv.org (2023).

[34] Verdicchio M Brancato V Cavaliere C Isgrò F Salvatore M Aiello M . A pathomic approach for tumor-infiltrating lymphocytes classification on breast cancer digital pathology images. Heliyon. (2023) 9:e14371. doi: 10.1016/j.heliyon.2023.e14371 36950640 PMC10025040

[35] Yosofvand M Khan SY Dhakal R Nejat A Moustaid-Moussa N Rahman RL . Automated detection and scoring of tumor-infiltrating lymphocytes in breast cancer histopathology slides. Cancers (Basel). (2023) 15:3635–49. doi: 10.3390/cancers15143635 37509295 PMC10377197

[36] van Dijk LAJBG de Groot THI . Automatic detection of tumour infiltrating lymphocytes in breast cancer whole slide images (2023). Stockholm: Stockholm University. Available online at: https://www.diva-portal.org/smash/get/diva2:1784341/FULLTEXT01.pdf (Accessed June 2, 2026).

[37] Jackson E Le F Lisbon JI Coleman M Burman J Wonderley A . Comprehensive experiments on breast cancer hematoxylin and eosin-stained images using UNet. In: Proceedings of the 2024 ACM Southeast Conference. New York, NY: ACM (2024). p. 121–8. doi: 10.1145/3603287.3651207

[38] Kim S Rakib Hasan K Ando Y Ko S Lee D Park NJY . Improving tumor-infiltrating lymphocytes score prediction in breast cancer with self-supervised learning. (2024) 14:90. doi: 10.3390/LIFE14010090 PMC11154396 38255705

[39] Liu S Amgad M More D Rathore MA Salgado R Cooper LAD . A panoptic segmentation dataset and deep-learning approach for explainable scoring of tumor-infiltrating lymphocytes. NPJ Breast Cancer. (2024) 10:52. doi: 10.1038/s41523-024-00663-1 38942745 PMC11213912

[40] Perera R Savas P Senanayake D Salgado R Joensuu H O’Toole S . Annotation-efficient deep learning for breast cancer whole-slide image classification using tumour infiltrating lymphocytes and slide-level labels. Commun Eng. (2024) 3:104. doi: 10.1038/s44172-024-00246-937880705

[41] Schirris Y Voorthuis R Opdam M Liefaard M Sonke GS Dackus G . ECTIL: Label-efficient computational tumour infiltrating lymphocyte (TIL) assessment in breast cancer: Multicentre validation in 2,340 patients with breast cancer. Lacet Digital Health. (2025) 7:100921. doi: 10.1016/j.landig.2025.100921 41381302

[42] Tafavvoghi M Bongo LA Delgado AB Shvetsov N Sildnes A Moi L . Automating tumor-infiltrating lymphocyte assessment in breast cancer histopathology images using QuPath: a transparent and accessible machine learning pipeline (2025). Available online at: https://arxiv.org/pdf/2504.16979 (Accessed July 23, 2026).

[43] Rijthoven M Aswolinskiy W Tessier L Salgado R van der Laak J Ciompi F . Analysis of computational tumor-infiltrating lymphocytes in breast cancer from the results of the TIGER challenge. Nat Commun. (2026) 17:6480. doi: 10.1038/s41467-026-72956-x 42140903 PMC13377072

[44] Yuan Y Failmezger H Rueda OM Raza Ali H Gräf S Chin SF . Quantitative image analysis of cellular heterogeneity in breast tumors complements genomic profiling. Sci Transl Med. (2012) 4:157ra143. doi: 10.1126/SCITRANSLMED.3004330 23100629

[45] Yuan Y . Modelling the spatial heterogeneity and molecular correlates of lymphocytic infiltration in triple-negative breast cancer. J R Soc Interface. (2015) 12:20141153. doi: 10.1098/RSIF.2014.1153 25505134 PMC4305416

[46] Krijgsman D van Leeuwen MB van der Ven J Almeida V Vlutters R Halter D . Quantitative whole slide assessment of tumor-infiltrating CD8-positive lymphocytes in ER-positive breast cancer in relation to clinical outcome. IEEE J BioMed Health Inform. (2020) 25:381–92. doi: 10.1109/JBHI.2020.3003475 32750943

[47] Lu Z Xu S Shao W Wu Y Zhang J Han Z . Deep-learning–based characterization of tumor-infiltrating lymphocytes in breast cancers from histopathology images and multiomics data. JCO Clin Cancer Inform. (2020) 4:480–90. doi: 10.1200/CCI.19.00126 32453636 PMC7265782

[48] Yu G Li X He T-F Gruosso T Zuo D Souleimanova M . Predicting relapse in patients with triple negative breast cancer (tnbc) using a deep-learning approach. Front Physiol. (2020) 11:511071. doi: 10.3389/fphys.2020.511071 33071806 PMC7538858

[49] Bai Y Cole K Martinez-Morilla S Ahmed FS Zugazagoitia J Staaf J . An open-source, automated tumor-infiltrating lymphocyte algorithm for prognosis in triple-negative breast cancer. Clin Cancer Res. (2021) 27:5557–65. doi: 10.1158/1078-0432.CCR-21-0325 34088723 PMC8530841

[50] Balkenhol MCA Ciompi F Świderska-Chadaj ŻChecktae van de Loo R Intezar M Otte-Höller I . Optimized tumour infiltrating lymphocyte assessment for triple negative breast cancer prognostics. Breast. (2021) 56:78–87. doi: 10.1016/j.breast.2021.02.007 33640523 PMC7933536

[51] Sun P He J Chao X Chen K Xu Y Huang Q . A computational tumor-infiltrating lymphocyte assessment method comparable with visual reporting guidelines for triple-negative breast cancer. EBioMedicine. (2021) 70:103492. doi: 10.1016/j.ebiom.2021.103492 34280779 PMC8318866

[52] Thagaard J Stovgaard ES Vognsen LG Hauberg S Dahl A Ebstrup T . Automated quantification of sTIL density with H&E-based digital image analysis has prognostic potential in triple-negative breast cancers. Cancers (Basel). (2021) 13:3050–65. doi: 10.3390/cancers13123050 34207414 PMC8235502

[53] Fassler DJ Torre-Healy LA Gupta R Hamilton AM Kobayashi S Van Alsten SC . Spatial characterization of tumor-infiltrating lymphocytes and breast cancer progression. Cancers (Basel). (2022) 14:2148–72. doi: 10.3390/cancers14092148 35565277 PMC9105398

[54] Liu H Kurc T . Deep learning for survival analysis in breast cancer with whole slide image data. Bioinformatics. (2022) 38:3629–37. doi: 10.1093/bioinformatics/btac381 35674341 PMC9272797

[55] Nederlof I Hajizadeh S Sobhani F Raza SEA AbdulJabbar K Harkes R . Spatial interplay of lymphocytes and fibroblasts in estrogen receptor-positive HER2-negative breast cancer. NPJ Breast Cancer. (2022) 8:56–65. doi: 10.1038/s41523-022-00416-y 35484275 PMC9051105

[56] Albusayli R Graham JD Pathmanathan N Shaban M Raza SEA Minhas F . Artificial intelligence‐based digital scores of stromal tumour‐infiltrating lymphocytes and tumour‐associated stroma predict disease‐specific survival in triple‐negative breast cancer. J Pathol. (2023) 260:32–42. doi: 10.1002/path.6061 36705810

[57] Fernandez G Zeineh J Prastawa M Scott R Madduri AS Shtabsky A . Analytical validation of the PreciseDx digital prognostic breast cancer test in early-stage breast cancer. Clin Breast Cancer. (2024) 24:93–102. doi: 10.1016/j.clbc.2023.10.008 38114366

[58] Makhlouf S Wahab N Toss M Ibrahim A Lashen AG Atallah NM . Evaluation of tumour infiltrating lymphocytes in luminal breast cancer using artificial intelligence. Br J Cancer. (2023) 129:1747–58. doi: 10.1038/s41416-023-02451-3 37777578 PMC10667537

[59] Roostee S Ehinger D Jönsson M Staaf J Aine M . Automated image quantification of immunohistochemistry-stained immune cells in triple-negative breast cancer. Res Sq. (2023). doi: 10.21203/rs.3.rs-3138809/v1 39271910 PMC11399404

[60] Vidal JM Tsiknakis N Staaf J Bosch A Ehinger A Nimeus E . The analytical and clinical validity of AI algorithms to score TILs in TNBC: can we use different machine learning models interchangeably? EClinicalMedicine. (2024) 78:102928. doi: 10.1016/J.ECLINM.2024.102928 39634035 PMC11615110

[61] Albalawi ES Qayyum J Qayyum J . Artificial intelligence based quantification of T lymphocyte infiltrate predicts prognosis in high grade breast cancer using deep learning and statistical validation. Discover Oncol. (2025) 16:2213. doi: 10.1007/S12672-025-04185-5 41353687 PMC12722604

[62] Ali HR Dariush A Provenzano E Bardwell H Abraham JE Iddawela M . Computational pathology of pre-treatment biopsies identifies lymphocyte density as a predictor of response to neoadjuvant chemotherapy in breast cancer. Breast Cancer Res. (2016) 18:21. doi: 10.1186/S13058-016-0682-8 26882907 PMC4755003

[63] Ali HR Dariush A Thomas J Provenzano E Dunn J Hiller L . Lymphocyte density determined by computational pathology validated as a predictor of response to neoadjuvant chemotherapy in breast cancer: secondary analysis of the ARTemis trial. Ann Oncol. (2017) 28:1832–5. doi: 10.1093/ANNONC/MDX266 28525534 PMC5834010

[64] Abe N Matsumoto H Takamatsu R Tamaki K Takigami N Uehara K . Quantitative digital image analysis of tumor-infiltrating lymphocytes in HER2-positive breast cancer. Virchows Arch. (2020) 476:701–9. doi: 10.1007/s00428-019-02730-6 31873876

[65] Li F Yang Y Wei Y He P Chen J Zheng Z . Deep learning-based predictive biomarker of pathological complete response to neoadjuvant chemotherapy from histological images in breast cancer. J Transl Med. (2021) 19:1–13. doi: 10.1186/S12967-021-03020-Z 34399795 PMC8365907

[66] Li B Li F Liu Z Xu FP Ye G Li W . Deep learning with biopsy whole slide images for pretreatment prediction of pathological complete response to neoadjuvant chemotherapy in breast cancer:A multicenter study. Breast. (2022) 66:183–90. doi: 10.1016/J.BREAST.2022.10.004 36308926 PMC9619175

[67] Li F Yang Y Wei Y Zhao Y Fu J Xiao X . Predicting neoadjuvant chemotherapy benefit using deep learning from stromal histology in breast cancer. NPJ Breast Cancer. (2022) 8:1–11. doi: 10.1038/s41523-022-00491-1 36418332 PMC9684483

[68] Aswolinskiy W Munari E Horlings HM Mulder L Bogina G Sanders J . PROACTING: predicting pathological complete response to neoadjuvant chemotherapy in breast cancer from routine diagnostic histopathology biopsies with deep learning. Breast Cancer Res. (2023) 25:1–15. doi: 10.1186/S13058-023-01726-0 37957667 PMC10644597

[69] Choi S Cho SI Jung W Lee T Choi SJ Song S . Deep learning model improves tumor-infiltrating lymphocyte evaluation and therapeutic response prediction in breast cancer. NPJ Breast Cancer. (2023) 9:1–13. doi: 10.1038/s41523-023-00577-4 37648694 PMC10469174

[70] Bhattarai S Saini G Li H Seth G Fisher TB Janssen EAM . Predicting neoadjuvant treatment response in triple-negative breast cancer using machine learning. Diagnostics. (2024) 14:74–87. doi: 10.3390/DIAGNOSTICS14010074 38201383 PMC10871101

[71] Fisher TB Saini G Rekha TS Krishnamurthy J Bhattarai S Callagy G . Digital image analysis and machine learning-assisted prediction of neoadjuvant chemotherapy response in triple-negative breast cancer. Breast Cancer Res. (2024) 26:1–13. doi: 10.1186/S13058-023-01752-Y 38238771 PMC10797728

[72] Rasic D Stovgaard EIS Jylling AMB Salgado R Hartman J Rantalainen M . AI assessment of tumor-infiltrating lymphocytes on routine H&E-slides as a predictor of response to neoadjuvant therapy in breast cancer—a real-world study. Virchows Arch. (2025), 1–12. doi: 10.1007/S00428-025-04283-3 41055693

[73] Liang H Zhu H Wang L Yu J Feng R Yan X . AI-driven spatial analysis of tumor-infiltrating lymphocytes predicts chemo-immunotherapy response in triple-negative breast cancer. Front Immunol. (2026) 17:1797753. doi: 10.3389/fimmu.2026.1797753 PMC13128803 42079614

[74] Schlam I Tang G AbdulJabbar K Yan H Gu X Freeman TJ . AI-powered and manual assessment of tumor-infiltrating lymphocytes in early HER2-positive breast cancer in NSABP B-41. NPJ Breast Cancer. (2026) 12(1):98. doi: 10.1038/S41523-026-00961-W 42135347 PMC13415812

[75] Xie T Ai S Liu C Luo B Yan H Zhang H . Focal hotspot and diffuse immune subtypes of tumor-infiltrating lymphocytes: AI-powered spatial clustering classification and its clinical relevance to HER2 expression in triple-negative breast cancer. J Trans Med 2025. (2025) 24(1):111. doi: 10.1186/S12967-025-07608-7 41437085 PMC12836918

[76] Denkert C Wienert S Poterie A Loibl S Budczies J Badve S . Standardized evaluation of tumor-infiltrating lymphocytes in breast cancer: Results of the ring studies of the international immuno-oncology biomarker working group. Mod Pathol. (2016) 29:1155–64. doi: 10.1038/modpathol.2016.109 27363491

[77] Loi S Michiels S Adams S Loibl S Budczies J Denkert C . The journey of tumor-infiltrating lymphocytes as a biomarker in breast cancer: clinical utility in an era of checkpoint inhibition. Ann Oncol. (2021) 32:1236–44. doi: 10.1016/J.ANNONC.2021.07.007 34311075

[78] Baharun NB Adam A Zailani MAH Rajpoot NM Xu Q Zin RRM . Automated scoring methods for quantitative interpretation of Tumour infiltrating lymphocytes (TILs) in breast cancer: a systematic review. BMC Cancer. (2024) 24:1202. doi: 10.1186/S12885-024-12962-8 39350098 PMC11440723

[79] Van Calster B McLernon DJ Van Smeden M Wynants L Steyerberg EW Bossuyt P . Calibration: the Achilles heel of predictive analytics. BMC Med. (2019) 17:230. doi: 10.1186/S12916-019-1466-7 31842878 PMC6912996

[80] Vickers AJ Elkin EB . Decision curve analysis: a novel method for evaluating prediction models. Med Decis Making. (2006) 26:565. doi: 10.1177/0272989X06295361 17099194 PMC2577036

[81] Tellez D Litjens G Bándi P Bulten W Bokhorst JM Ciompi F . Quantifying the effects of data augmentation and stain color normalization in convolutional neural networks for computational pathology. Med Image Anal. (2019) 58:101544. doi: 10.1016/j.media.2019.101544 31466046

[82] Tolaney SM Garrett-Mayer E White J Blinder VS Foster JC Amiri-Kordestani L . Updated standardized definitions for efficacy end points (STEEP) in adjuvant breast cancer clinical trials: STEEP version 2.0. J Clin Oncol. (2021) 39:2720–31. doi: 10.1200/JCO.20.03613 34003702 PMC10166345

[83] McShane LM Altman DG Sauerbrei W Taube SE Gion M Clark GM . Reporting recommendations for tumor marker prognostic studies (REMARK). J Natl Cancer Inst. (2005) 97:1180–4. doi: 10.1093/JNCI/DJI237 16106022

[84] Eiamprapaporn P Mata CV Nandani R Susiriwatananont T Knutson KL Chumsri S . Tumor-infiltrating lymphocytes in triple-negative breast cancer: prognostic significance, predictive value, and emerging directions for clinical implementation. Cancers (Basel). (2026) 18:1588. doi: 10.3390/CANCERS18101588 42192948 PMC13204212

[85] Alharbi S Qasem FF Talebi MT Omran NE Hamoudi R Harati R . Immunotherapy approaches for the treatment of triple-negative breast cancer. Cancers (Basel). (2026) 18:464. doi: 10.3390/CANCERS18030464 41681936 PMC12897434

[86] Seymour L Bogaerts J Perrone A Ford R Schwartz LH Mandrekar S . iRECIST: guidelines for response criteria for use in trials testing immunotherapeutics. Lancet Oncol. (2017) 18:e143–52. doi: 10.1016/S1470-2045(17)30074-8 28271869 PMC5648544

[87] Chen RJ Ding T Lu MY Williamson DFK Jaume G Song AH . Towards a general-purpose foundation model for computational pathology. Nat Med. (2024) 30:850–62. doi: 10.1038/s41591-024-02857-3 38504018 PMC11403354

[88] Xu H Usuyama N Bagga J Zhang S Rao R Naumann T . A whole-slide foundation model for digital pathology from real-world data. Nature. (2024) 630:181–8. doi: 10.1038/s41586-024-07441-w 38778098 PMC11153137

[89] Lu MY Chen B Williamson DFK Chen RJ Liang I Ding T . A visual-language foundation model for computational pathology. Nat Med. (2024) 30:863–74. doi: 10.1038/s41591-024-02856-4 38504017 PMC11384335

[90] Tejani AS Klontzas ME Gatti AA Mongan JT Moy L Park SH . Checklist for artificial intelligence in medical imaging (CLAIM): 2024 update. Radiol Artif Intell. (2024) 6. doi: 10.1148/RYAI.240300 38809149 PMC11304031

[91] Norgeot B Quer G Beaulieu-Jones BK Torkamani A Dias R GianFrancesco M . Minimum information about clinical artificial intelligence modeling: the MI-CLAIM checklist. Nat Med. (2020) 26:1320–4. doi: 10.1038/S41591-020-1041-Y 32908275 PMC7538196

[92] Sounderajah V Guni A Liu X Collins GS Karthikesalingam A Markar SR . The STARD-AI reporting guideline for diagnostic accuracy studies using artificial intelligence. Nat Med. (2025) 31:3283–9. doi: 10.1038/s41591-025-03953-8 40954311

[93] Cortazar P Zhang L Untch M Mehta K Costantino JP Wolmark N . Pathological complete response and long-term clinical benefit in breast cancer: The CTNeoBC pooled analysis. Lancet. (2014) 384:164–72. doi: 10.1016/S0140-6736(13)62422-8 24529560

[94] Vasey B Nagendran M Campbell B Clifton DA Collins GS Denaxas S . Reporting guideline for the early stage clinical evaluation of decision support systems driven by artificial intelligence: DECIDE-AI. BMJ. (2022) 377:e070904. doi: 10.1136/BMJ-2022-070904 35584845 PMC9116198

[95] Moons KGM Damen JAA Kaul T Hooft L Andaur Navarro C Dhiman P . PROBAST+AI: an updated quality, risk of bias, and applicability assessment tool for prediction models using regression or artificial intelligence methods. BMJ. (2025) 388. doi: 10.1136/BMJ-2024-082505 40127903 PMC11931409

[96] Saldanha OL Quirke P West NP James JA Loughrey MB Grabsch HI . Swarm learning for decentralized artificial intelligence in cancer histopathology. Nat Med. (2022) 28:1232–9. doi: 10.1038/s41591-022-01768-5 35469069 PMC9205774

[97] Begoli E Bhattacharya T Kusnezov D . The need for uncertainty quantification in machine-assisted medical decision making. Nat Mach Intell. (2019) 1:20–3. doi: 10.1038/S42256-018-0004-137880705

