# Supplementary material for: Machine learning-based assessment of tumor-infiltrating lymphocytes in breast cancer histopathology: a systematic review and evidence-gap analysis of translational readiness
Source: Front Oncol. 2026 Oct 26;16:1908349. doi: 10.3389/fonc.2026.1908349 (PMC13745231; doi:10.3389/fonc.2026.1908349)
Supplement: Supplementary file 1 [file Table1.docx]

**Table S1. Search Strategy Query**

| PubMed  Filters   1) Article language: English 2) Species: Human | (  ("Breast Neoplasms"[Mesh]  OR breast cancer[tiab]  OR breast carcinoma[tiab]  OR mammary cancer[tiab])  )  AND  (  ("Tumor-Infiltrating Lymphocytes"[Mesh]  OR tumor infiltrating lymphocytes[tiab]  OR TIL[tiab]  OR TILs[tiab]  OR stromal TIL[tiab]  OR intratumoral TIL[tiab])  )  AND  (  ("Machine Learning"[Mesh]  OR "Artificial Intelligence"[Mesh]  OR "Deep Learning"[Mesh]  OR machine learning[tiab]  OR deep learning[tiab]  OR artificial intelligence[tiab]  OR neural network*[tiab]  OR convolutional neural network*[tiab]  OR CNN[tiab]  OR radiomics[tiab]  OR computational pathology[tiab])  ) |
| --- | --- |
| Scopus  Filters:   1) Document type: Article 2) Language: Limited to English | ( TITLE-ABS-KEY("breast cancer" OR "breast carcinoma" OR "mammary cancer" OR "breast neoplasm*"))  AND  ( TITLE-ABS-KEY("tumor infiltrating lymphocyte*" OR "tumour infiltrating lymphocyte*" OR TIL OR TILs OR "stromal TIL*" OR "intratumoral TIL*" OR "intra-tumoral TIL*"))  AND  ( TITLE-ABS-KEY("machine learning" OR "deep learning" OR "artificial intelligence" OR "neural network*" OR "convolutional neural network*" OR CNN OR "computational pathology")) |
| ScienceDirect (no additional filters) | (  TITLE-ABS-KEY("breast cancer" OR "breast carcinoma")  )  AND  (  TITLE-ABS-KEY("tumor infiltrating lymphocytes" OR "tumour infiltrating lymphocytes" OR TIL )  )  AND  (  TITLE-ABS-KEY("machine learning" OR "deep learning" OR "artificial intelligence")  ) |
| Web of Science  Filters:   1) Document type: Article 2) Language: Limited to English | TS=(  ("breast cancer" OR "breast carcinoma" OR "mammary cancer" OR "breast neoplasm*")  AND  ("tumor infiltrating lymphocyte*" OR "tumour infiltrating lymphocyte*" OR TIL OR TILs OR "stromal TIL*" OR "intratumoral TIL*" OR "intra-tumoral TIL*")  AND  ("machine learning" OR "deep learning" OR "artificial intelligence" OR "neural network*" OR "convolutional neural network*" OR CNN OR radiomics OR "computational pathology")  ) |
| Google Scholar  Search in title | "breast cancer" AND ("tumor infiltrating lymphocytes" OR TIL OR TILs) AND ("machine learning" OR "deep learning" OR "artificial intelligence" OR "computational pathology") |

**Table S2. Description of the included studies**

| **Study, country** | **Dataset and sample** | **Specimen / endpoint** | **Architecture / explainability** | **Validation / performance** | **Clinical applicability / reproducibility / overlap** |
| --- | --- | --- | --- | --- | --- |
| Detection classification |  |  |  |  |  |
| Basavanhally et al., 2010, USA (25) | Private/internal; Single-center; patients: 12; slides/images: 41; tiles/annotations: 41 image samples | Surgical resection; H&E; Digitized histopathology images; | Region growing + Markov random field; Voronoi, Delaunay and minimum-spanning-tree features; graph embedding + SVM; Explainability: Explicit spatial/cell/tissue features or maps | Repeated three-fold cross-validation (>100 trials); external: No; split: Image Classification accuracy: >90% for high versus low lymphocytic infiltration | Automated/assisted TIL detection, scoring, quality control or research annotation Code: No; data: No |
| Turkki et al., 2016, Finland/Sweden (26) | Private/internal; Single-center; patients: 20; slides/images: Paired consecutive H&E and CD45-stained sections from 20 patients; tiles/annotations: 123,442 labelled superpixels | Formalin-fixed paraffin-embedded primary tumour; H&E and CD45 IHC; Whole-slide image; timing: Primary surgery | Pretrained CNN feature extractor with support-vector-machine classification of immune-rich regions; learning: Antibody-supervised; pretraining: Generic pretrained CNN | Internal cross-validation and pathologist agreement assessment; external: No; split: Superpixel/image F1 score; agreement: CNN F1 0.94 versus texture F1 0.88; 90% agreement, κ=0.79 | Automated/assisted TIL detection, scoring, quality control or research annotation Code: No; data: No |
| Amgad et al., 2019, USA (27) | Private/internal; Single-center; patients: Not reported; slides/images: 120; tiles/annotations: 14 annotated slides; 5 held out; 1024x1024 tiles | Surgical resection; H&E; Whole-slide image; | FCN-8 / VGG16 fully convolutional network with joint region and nucleus labels; learning: Supervised; pretraining: ImageNet Explainability: Explicit spatial/cell/tissue features or maps | Internal split only; external: No; split: Slide Dice; AUC; Correlation: Dice overall 0.78; tumor 0.83; fibroblast 0.77; lymphocyte AUC 0.89; c-sTIL vs consensus R=0.73 | Automated/assisted TIL detection, scoring, quality control or research annotation Code: No; data: No |
| Le et al., 2020, USA (28) | Public; TCGA-BRCA; Public dataset only; patients: TCGA-BRCA cases; slides/images: 1090; tiles/annotations: Superpatch/tile-based CNN inference | Surgical resection; H&E; Whole-slide image; | ResNet-34; VGG-16; Inception v4 pipelines for tumor and TIL maps; learning: Supervised; pretraining: ImageNet Explainability: Explicit spatial/cell/tissue features or maps | Internal split only; external: No; split: Slide AUC; survival HR: Open maps for 1090 TCGA invasive breast cancer images; CNN metrics and survival associations reported | Automated/assisted TIL detection, scoring, quality control or research annotation Code: No; data: Yes Overlap: Potential public-cohort reuse. |
| Lee et al., 2021, USA (29) | Public; TCGA-BRCA; Public dataset only; slides/images: TCGA/BRCA demonstration; ; tiles/annotations: Superpixel/annotation examples | Surgical resection; H&E; Whole-slide image; | HistomicsML2 interactive learn-by-example classifier; learning: Supervised; pretraining: Not applicable Explainability: Explicit spatial/cell/tissue features or maps | Internal split only; external: No; split: Patch/tile Accuracy; F1: reported for user-defined validation annotations; a single breast-cancer TIL performance estimate was NR. | Automated/assisted TIL detection, scoring, quality control or research annotation Code: Yes; data: Yes Overlap: Potential public-cohort reuse |
| Narayanan et al., 2021, UK/USA (30) | Private/internal; multi-center; patients: Pure DCIS and adjacent DCIS cohort; tiles/annotations: Duct-level and TIL spatial units | Surgical resection; H&E; Whole-slide image; | UNMaSk pipeline: IM-Net duct detection and DRDIN TIL mapping; Explainability: Explicit spatial/cell/tissue features or maps | Internal split only; external: No; split: Region/ROI Detection/segmentation metrics: Automated duct/TIL spatial mapping; exact metrics require verification | Automated/assisted TIL detection, scoring, quality control or research annotation Code: Yes; data: Yes |
| Negahbani et al., 2021, Iran (31) | Private/internal; SHIDC-BC-Ki-67; Single-center; slides/images: SHIDC-BC-Ki-67 dataset; tiles/annotations: Annotated Ki-67/TIL cell dataset | Surgical resection:; IHC; Region of interest; | PathoNet deep neural-network backend | Internal split only; external: No; split: Patch/tile F1: Outperforms benchmark on harmonic mean/F1; exact value NR | Automated/assisted TIL detection, scoring, quality control or research annotation Code: Yes; data: Yes |
| Schirris et al., 2021, Netherlands (32) | Private/internal; Single-center; slides/images: WSI-level sTIL score dataset; tiles/annotations: 512x512 tiles at 0.5 mpp | Surgical resection; H&E; Whole-slide image; | WeakSTIL: SSL feature extractor + MIL regressor; learning: Self-supervised pretraining + supervised; pretraining: Histology-specific self-supervised Explainability: Explicit spatial/cell/tissue features or maps | Cross-validation; external: No; split: Patient AUC; R-squared: sTIL-high vs low AUC 0.89 ± 0.05; R2 0.45 ± 0.15 vs expert pathologist | Automated/assisted TIL detection, scoring, quality control or research annotation Code: No; data: Yes |
| Shephard et al., 2023, UK (33) | Challenge dataset; TIGER challenge cohort 195; TCGA-BRCA,; Public dataset; RUMC and JB – private dataset patients: slides/images: TIGER challenge training/leaderboard data; tiles/annotations: Tissue segmentation/TIL detection tiles | Surgical resection; H&E; Whole-slide image; | Efficient-UNet for tumor/stroma segmentation and TIL detection;  Explainability: Explicit spatial/cell/tissue features or maps | Internal + external validation; external: Yes; split: Slide Dice; F1; C-index: Internal cross-validation: mean Dice 0.742; F1 0.702; recall 0.774; precision 0.642 | Automated/assisted TIL detection, scoring, quality control or research annotation Code: Yes; data: Yes Overlap: Potential public-cohort reuse; overlap |
| Verdicchio et al., 2023, Italy (34) | Challenge dataset; TIGER challenge cohort 195; TCGA-BRCA,; Public dataset; RUMC and JB – private dataset patients: slides/images: Not reported; tiles/annotations: 1037 | Surgical resection; H&E; Whole-slide image; | Watershed segmentation + 71 pathomic features + RF/SVM/logistic-type classifiers; Explainability: Handcrafted features with interpretable feature definitions | Cross-validation; external: No; split: Region/ROI AUC: Best Random Forest ROC-AUC 0.86 | Automated/assisted TIL detection, scoring, quality control or research annotation Code: No; data: Yes. Overlap: potential overlap |
| Yosofvand et al., 2023, USA (35) | Private/internal; Single-center; slides/images: 63; tiles/annotations: Annotated TIL/stroma image regions | Surgical resection; H&E; Whole-slide image; | U-Net for stroma detection + Mask R-CNN for TIL detection + GUI; Explainability: Not reported or limited | Internal split only; external: No; split: Slide Accuracy; correlation: Stroma U-Net accuracy reported as 98%; TIL detection/scoring compared with pathologists | Automated/assisted TIL detection, scoring, quality control or research annotation Code: No; data: No |
| van Dijk et al., 2023, Sweden (36) | Challenge dataset; TIGER; Public dataset only; patients: Not applicable/challenge dataset; slides/images: TIGER data; tiles/annotations: YOLOv5 object detection tiles | Surgical resection; H&E; Whole-slide image; | YOLOv5m object detection model; learning: Transfer learning; pretraining: Other Explainability: Not reported or limited | Internal split only; external: No; split: Patch/tile Precision; recall: Precision 0.668; recall 0.621 | Automated/assisted TIL detection, scoring, quality control or research annotation Code: Yes; data: Yes Overlap: Potential public-cohort reuse; |
| Jackson et al., 2024, USA (37) | Public; BCSS for experiment H&E/TIL datasets; tiles/annotations: Tumor/stroma/TIL segmentation tiles | Surgical resection; H&E; Whole-slide image; | Modified U-Net with input-size experiments and neural-style guided augmentation;  Explainability: Not reported or limited | Internal split only; external: No; split: Patch/tile Dice; segmentation performance: Modified U-Net performed well; exact metric values require verification from tables | Automated/assisted TIL detection, scoring, quality control or research annotation Code: No; data: Yes |
| Kim et al., 2024, South Korea (38) | Challenge/public; TIGER challenge cohort;; Multi-center challenge dataset; patients: Study-specific TIGER cohorts; patient count NR; slides/images: 82 WSIs for TIL-score prediction; 151 BCSS images and 1,879 ROI images for tissue tasks; tiles/annotations: Approximately 3,195 downstream images plus large SSL pretraining corpus | Biopsy/resection challenge data; H&E; Whole-slide image and ROI patches; | SimCLR and SwAV ResNet-18 pretraining transferred to DeepLabv3/U-Net tissue segmentation and lymphocyte detection; learning: Self-supervised + supervised fine-tuning; pretraining: SimCLR/SwAV pathology pretraining | Five-fold cross-validation on TIGER-derived task splits; external: No; split: Image/ROI Dice; FROC; Pearson correlation: Best tissue Dice 0.897; lymphocyte FROC 0.682; TIL-score correlation r=0.718 | Automated/assisted TIL detection, scoring, quality control or research annotation Code: No; data: Yes Overlap potential overlap |
| Liu et al., 2024, USA/Belgium/Australia (39)  . | Public; PanopTILs; BCSS; NuCLS; Multi-center public data; patients: 151; slides/images: PanopTILs derived from 151 patients; tiles/annotations: 814,886 annotated nuclei | Surgical resection; H&E; Whole-slide images and annotated regions; timing: Primary surgery | MuTILs joint multi-resolution tissue-region and nucleus segmentation/classification; learning: Supervised; pretraining: ImageNet/pathology components Explainability: Explicit spatial/cell/tissue features or maps | Internal-external five-fold cross-validation by hospital plus outcome analysis; external: Yes; split: Hospital/patient AUROC; Dice; correlation: Lymphocyte AUROC 0.93; tumour-associated stroma Dice 0.81; computational versus visual TIL r=0.58–0.61 | Automated/assisted TIL detection, scoring, quality control or research annotation Code: Yes; weights: Yes; data: Yes Overlap: Potential public-cohort reuse |
| Perera et al., 2024, Australia (40) | Public + private; CLEOPATRA; FinHER; TCGA-BRCA; TIGER; Multi-center/multi-cohort; patients: Study-specific slide cohorts; slides/images: CLEOPATRA 747; FinHER 651; TCGA 695; independent TIGER test 82; tiles/annotations: Patch embeddings across WSIs | Biopsy and surgical resection; H&E; Whole-slide image; timing: Mixed | ANSAC annotation-efficient segmentation and attention-based WSI classifier; learning: Weakly supervised; pretraining: Pretrained CNN feature extraction Explainability: Attention maps plus spatial TIL features | Held-out cohort tests plus independent TIGER evaluation; external: Yes; split: Patient/slide AUROC: Internal AUROCs approximately 0.86–0.95; independent TIGER AUROC up to 0.851 | Automated/assisted TIL detection, scoring, quality control or research annotation Code: Yes; data: Partial Overlap: Potential public-cohort reuse |
| Schirris et al., 2025, Netherlands (41) | Public + private; TCGA-BRCA; BASIS; N4+; MATADOR; ICGC, TRAIN-2; PARADIGM; Multi-center; six cohorts including randomized trials; patients: 2340; slides/images: 2,354 WSIs across six cohorts; tiles/annotations: Foundation-model patch embeddings from WSIs | Biopsy and surgical resection; H&E; Whole-slide image; timing: Mixed | RetCCL pathology foundation-model embeddings with gated-attention MIL regression; learning: Weakly supervised; pretraining: Pathology foundation model Explainability: Attention maps plus spatial TIL features | Five heterogeneous external cohorts; independent PARADIGM test set; external: Yes; split: Patient/cohort Correlation; AUROC; hazard ratio: External r=0.54–0.74 and AUROC=0.80–0.94; PARADIGM r=0.69, AUROC=0.85; OS HR per 10% increase 0.86 (0.78–0.94) | Automated/assisted TIL detection, scoring, quality control or research annotation Code: Yes; weights: Yes; data: Partial Overlap: Distinct from WeakSTIL 2021; later foundation-model/MIL approach and different cohorts. |
| Tafavvoghi et al., 2025, Norway (42) | Public + private; TIGER; CAMO; Multi-center external test; patients: CAMO external test: 53 eligible patients; slides/images: TIGER WSIROIS 195; TIGER WSITILS 82; CAMO external 53 WSIs; tiles/annotations: Approximately 34,000 balanced cell examples plus 70 external ROIs | Biopsy/resection; H&E; Whole-slide images and ROIs; timing: Mixed | QuPath random-forest pixel classifier, pretrained StarDist cell detector and random-forest TIL classifier; learning: Supervised; pretraining: Pretrained StarDist | TIGER development and independent CAMO external test; external: Yes; split: Patient/WSI Accuracy; Cohen κ: External cell-classification accuracy 0.96; external WSI TIL-category κ=0.71 | Automated/assisted TIL detection, scoring, quality control or research annotation Code: No; weights: Partial; data: Partial Overlap: Potential overlap; |
| van Rijthoven et al., 2026, Netherlands/International (43) | Challenge dataset; TIGER challenge cohort;; TCGA-BRCA,; Public dataset; RUMC and JB – private dataset patients: TIGER challenge cohort 195; tiles/annotations: SEGTIGER 286 ROIs/195 WSIs; DETTIGER 1,879 ROIs/168 WSIs  FinHER | Biopsy and surgical resection; H&E; selected restained IHC; Whole-slide images and ROIs; timing: Pretreatment and primary surgery | Nine open-source TIGER computational TIL models spanning segmentation, detection and score-estimation pipelines; learning: Multiple; pretraining: Multiple Explainability: Explicit spatial/cell/tissue features or maps | Completely independent post-challenge multicentre clinical validation; external: Yes; split: Cohort/patient Correlation; AUROC; C-index: Mean model–pathologist correlation ρ=0.61; pCR AUROC ranges TNBC 0.42–0.87 and HER2+ 0.52–0.78; prognostic C-index approximately 0.71–0.74 | Automated/assisted TIL detection, scoring, quality control or research annotation Code: Yes; weights: Yes; data: Yes Overlap: potential overlap |
| Outcome Prognostication |  |  |  |  |  |
| Yuan et al., 2012, UK/Canada (44) | Public/private; METABRIC, Multi-center; patients: 564; slides/images: 323 discovery + 241 validation tissue sections; tiles/annotations: Cell-level classification and spatial statistics | Surgical resection; H&E; Digitized tissue sections; timing: Treatment-naive/primary surgery Clinical endpoint: Survival in ER-negative breast cancer; | Pathologist-trained SVM cell classifier, spatial K-function features and integrated image–gene-expression model; learning: Explainability: Explicit spatial/cell/tissue features or maps | Independent discovery and validation cohorts; external: Yes; split: Patient Hazard ratio; prediction accuracy: Image lymphocyte proportion HR 0.27 (0.08–0.88) in discovery and 0.40 (0.16–0.98) in validation; integrated prediction approximately 86% | Risk stratification/prognostic biomarker development; prospective utility not established Code: Yes; data: Yes |
| Yuan et al., 2015, UK (45) | Public/private; METABRIC, Multi-center; patients: 181; slides/images: 181 whole-tumour H&E sections (89 and 92 patients); tiles/annotations: Cell-level spatial measurements | Surgical resection; H&E; Whole-tumour section images; timing: Primary surgery Clinical endpoint: Disease-specific survival; | Automated H&E cell classification plus spatial statistical modelling of intratumour lymphocyte ratio; Explainability: Explicit spatial/cell/tissue features or maps | Two independent cohorts with reciprocal discovery/validation analysis; external: Yes; split: Patient Hazard ratio: Intratumour lymphocyte ratio associated with disease-specific survival; validation HR 0.25 (0.09–0.69) | Risk stratification/prognostic biomarker development; prospective utility not established Code: Yes; data: Yes |
| Krijgsman et al., 2020, Netherlands (46) | Private/internal; Single-center; slides/images: Sequential HE and CD8 IHC slides; tiles/annotations: Spatial CD8 density maps | Surgical resection; H&E + IHC; Whole-slide image; timing: Adjuvant endocrine therapy cohort Clinical endpoint: Clinical outcome in tamoxifen-treated ER+ cohort; | Deep-learning tissue-type classification on HE + nucleus detection on CD8 IHC with image registration; Explainability: Explicit spatial/cell/tissue features or maps | Internal split only; external: No; split: Patient HR; correlation: Spatial CD8 density standard deviation in central tumor/invasive margin associated with clinical outcome; exact value NR | Risk stratification/prognostic biomarker development; prospective utility not established Code: No; data: No |
| Lu et al., 2020, USA/China (47) | Public; TCGA-BRCA; Public dataset only; patients: TCGA-BRCA patients with available WSIs and omics; slides/images: 1000; tiles/annotations: Cell-level TIL maps and 43 spatial TIL features | Surgical resection; H&E; Whole-slide image;  Clinical endpoint: Subtype-specific survival associations of 43 TIL spatial features; | Cascade-trained U-Net with ResNet18 encoder for lymphocyte detection; learning: Supervised; pretraining: ImageNet Explainability: Explicit spatial/cell/tissue features or maps | Internal split only; external: No; split: Patient F1; survival HR: U-Net lymphocyte detection recall 0.9536, precision 0.901, F1 0.9266; survival/omics associations reported | Risk stratification/prognostic biomarker development; prospective utility not established Code: No; data: Yes Overlap: Potential public-cohort reuse |
| Yu et al., 2020, USA/Canada (48) | Private/internal; multi-center; patients: 55; tiles/annotations: 64x64 image patches | Surgical resection; Immunofluorescence/IHC-like multiplex CD8/cytokeratin/DAPI; Region of interest; timing: Treatment-naive surgery Clinical endpoint: 3-year relapse / poor vs good outcome; | MXNet/deepflow patch classifier using CD8+ T-cell/cancer-cell spatial images;  Explainability: Explicit spatial/cell/tissue features or maps | Internal + external validation; external: Yes; split: Patient Accuracy; sensitivity/specificity: Predicted 3-year relapse likelihood; poor-outcome recall 1.00 and precision 0.50 in reported confusion matrix | Risk stratification/prognostic biomarker development; prospective utility not established Code: Yes; data: Yes |
| Bai et al., 2021, USA/Sweden (49) | Private/internal; multi-center; patients: 920 total (171 discovery; 749 validation); tiles/annotations: Cell-level classifier outputs | Surgical resection; H&E; Whole-slide image; timing: Primary surgical specimens Clinical endpoint: Overall survival in TNBC validation cohorts; | QuPath neural-network cell classifier for tumor cells, lymphocytes, fibroblasts and other cells;  Explainability: Explicit spatial/cell/tissue features or maps | Internal + external validation; external: Yes; split: Patient HR: Five machine TIL variables prognostic; adjusted Cox P≤0.0003; HRs reported with 95% CI | Risk stratification/prognostic biomarker development; prospective utility not established Code: No; data: Partial |
| Balkenhol et al., 2021, Netherlands (50) | Private/internal; Single center; patients: 94; slides/images: Subset from larger TNBC cohort; multiplex IHC and H&E sections; | Surgical resection; H&E + IHC; Whole-slide image; timing: Primary surgical specimens Clinical endpoint: Relapse-free survival and overall survival; | Advanced WSI analysis algorithms including CNN-based registration, tissue compartment segmentation and positive lymphocyte detection; Explainability: Explicit spatial/cell/tissue features or maps | Internal split only; external: No; split: Patient HR: TIL marker densities associated with RFS/OS; no marker clearly superior | Risk stratification/prognostic biomarker development; prospective utility not established Code: No; data: No |
| Sun et al., 2021, China (51) | Public + private; TCGA-BRCA; Multi-center; patients: 301 (Asian cohort n=184; Caucasian cohort n=117); tiles/annotations: Cell/nucleus-level outputs | Surgical resection; H&E; Whole-slide image; timing: Primary surgical specimens Clinical endpoint: Disease-free survival in TNBC; | Three deep neural networks for nuclei segmentation, nuclei classification and necrosis classification; Explainability: Explicit spatial/cell/tissue features or maps | Internal + external validation; external: Yes; split: Patient HR: aTIL associated with DFS; continuous HR=0.96 (95% CI 0.94–0.99); dichotomous HR=0.29 (95% CI 0.12–0.72) | Risk stratification/prognostic biomarker development; prospective utility not established Code: No; data: Partial Overlap: Potential public-cohort reuse |
| Thagaard et al., 2021, Denmark (52) | Private/internal; Single center; patients: 257; | Surgical resection; H&E; Whole-slide image; timing: Primary surgical specimens Clinical endpoint: Overall survival and prognostic potential of automated sTIL density; | H&E digital image analysis pipeline for automated sTIL density quantification; Explainability: Explicit spatial/cell/tissue features or maps | Internal split only; external: No; split: Patient HR: Automated sTIL density associated with prognosis; high concordance with manual scoring | Risk stratification/prognostic biomarker development; prospective utility not established Code: No; data: Yes |
| Fassler et al., 2022, USA (53) | Public + private; TCGA-BRCA; UNC CBCS; Multi-center; patients: TCGA-BRCA and UNC CBCS cohorts; 1098 patients; slides/images: TCGA and UNC WSIs; tiles/annotations: Patch/cell-level spatial features | Surgical resection; H&E; Whole-slide image; timing: Primary surgical specimens Clinical endpoint: Overall survival and progression-free interval; | Computer-vision/pathomics pipeline using TIL spatial abundance and forest/desert spatial features; Explainability: Explicit spatial/cell/tissue features or maps | Internal + external validation; external: Yes; split: Patient HR: Spatial TIL features associated with OS/PFI; high-risk spatial features linked to shorter PFI | Risk stratification/prognostic biomarker development; prospective utility not established Code: No; data: Yes Overlap: Potential public-cohort reuse |
| Liu et al., 2022, USA (54) | Public; TCGA-BRCA; Public dataset only; patients: 978 TCGA-BRCA cases; slides/images: 978 diagnostic WSIs; tiles/annotations: Multi-resolution WSI patches plus tumor/TIL/nucleus maps | Surgical resection; H&E; Whole-slide image; timing: Diagnostic slides Clinical endpoint: Breast cancer survival analysis; | Multi-resolution deep survival model integrating RGB images with tumor, lymphocyte and nuclear segmentation maps; Explainability: Explicit spatial/cell/tissue features or maps | Internal split only; external: No; split: Patient C-index: C-index 0.706 for image+NTL model; 0.773 when clinical features added; bootstrap 95% CIs reported | Risk stratification/prognostic biomarker development; prospective utility not established Code: Yes; data: Yes Overlap: Potential public-cohort reuse; |
| Nederlof et al., 2022, Netherlands/UK (55) | Public + private; ICGC; METABRIC; Multi-center; patients: 179 discovery; 630 validation; slides/images: ICGC and METABRIC H&E slides; tiles/annotations: Cell/region spatial-pattern features | Surgical resection; H&E; Whole-slide image; timing: Primary surgical specimens Clinical endpoint: Overall survival and clinical impact of spatial lymphocyte/fibroblast patterns; | Deep neural network to locate tumor, TILs and fibroblasts with density estimation spatial analysis; Explainability: Explicit spatial/cell/tissue features or maps | Internal + external validation; external: Yes; split: Patient HR: Spatial fibroblast/TIL mixing associated with prognosis; e.g., HR 0.464 for spatial mixing | Risk stratification/prognostic biomarker development; prospective utility not established Code: Yes; data: Yes |
| Albusayli et al., 2023, UK/Australia (56) | Public + private; AUBC; Multi-center; patients: 429 total (AUBC n=318; TCGA n=111); slides/images: 429 WSIs; 46 TCGA slides excluded before final TCGA n=111; tiles/annotations: Spatial sTME features | Surgical resection; H&E; Whole-slide image; timing: Primary surgical specimens Clinical endpoint: Disease-specific survival in TNBC; | Deep learning tissue-region segmentation into tumor, stroma and lymphocytes; Digi-sTILs/Digi-TAS features; Explainability: Explicit spatial/cell/tissue features or maps | Internal + external validation; external: Yes; split: Patient C-index: Digi-sTILs C-index 0.65; Digi-TAS C-index 0.60 on TCGA; Digi-sTILs + node status C-index 0.76 | Risk stratification/prognostic biomarker development; prospective utility not established Code: Yes; data: Partial Overlap: Potential public-cohort reuse |
| Fernandez et al., 2023, USA (57) | Private/internal; Single-center; patients: Analytical precision subset: 8 patients; source clinical archive 2004-2016; tiles/annotations: 237,661 ROIs for lymphocyte-like patch classification; 161,801 train and 75,860 test | Surgical resection; H&E; Whole-slide image; timing: At diagnosis / early-stage invasive breast cancer Clinical endpoint: Early-stage breast cancer recurrence within 6 years / disease-free survival risk score; | Morphology feature array with CNN/ResNet lymphocyte detection and proprietary composite risk score; Explainability: Explicit spatial/cell/tissue features or maps | Internal split only; external: No; split: Slide Precision; recall; F1; reproducibility CV: Lymphocyte detection precision/recall 0.91/0.93; risk-score repeatability/reproducibility CV <2% | Risk stratification/prognostic biomarker development; prospective utility not established Code: No; data: No |
| Makhlouf et al., 2023, UK (58) | Public/private; Nottingham cohort: and multi-center private cohort, and BCSS; patients: 2231 total (1572 training/discovery; 659 test; external cohort also described); tiles/annotations: Cell-level TIL counts and spatial features | Surgical resection; H&E; Whole-slide image; timing: Primary surgical specimens Clinical endpoint: Long-term outcome in luminal early-stage breast cancer; | Supervised deep learning model for H&E WSI TIL detection and spatial co-occurrence analysis;  Explainability: Explicit spatial/cell/tissue features or maps | Internal + external validation; external: Yes; split: Patient HR: AI TIL counts/spatial co-occurrence associated with outcomes; tTILs independent predictor of worse outcome | Risk stratification/prognostic biomarker development; prospective utility not established Code: No; data: No |
| Roostee et al., 2023, Sweden (59) | Private/internal; SCAN-B; Single-center; patients: 218; slides/images: TMA cores stained for p53, CD3, CD4, CD8, CD20, CD68, FOXP3, PD-L1; tiles/annotations: Cell counts by marker | TMA; IHC; Tissue microarray; timing: Primary surgical specimens Clinical endpoint: Patient outcome patterns by immune-marker cell counts and molecular features; | CNN-based instance segmentation/image-analysis pipeline; Explainability: Not reported or limited | Internal split only; external: No; split: Patient HR: IHC immune-cell counts correlated with pathology review/RNA-seq signatures and known prognostic patterns | Risk stratification/prognostic biomarker development; prospective utility not established Code: Yes; data: Yes |
| Martínez Vidal et al., 2024, Sweden/USA (60) | Private/internal; Multi-center; patients: 79 development/analytical cohort; 215 independent prospective cohort; slides/images: 92 slides in development cohort; Swedish validation cohort slides; tiles/annotations: ROI/cell classifier outputs across 10 AI models | Surgical resection; H&E; Whole-slide image; timing: Diagnostic tissue slides Clinical endpoint: Invasive disease-free survival with median 4-year follow-up; | Ten QuPath-based AI TIL scoring models with different training strategies; learning: Explainability: Explicit spatial/cell/tissue features or maps | Internal + external validation; external: Yes; split: Patient HR: Analytical validity Spearman r=0.63–0.73; prognostic HR 0.40–0.47 in external cohort | Risk stratification/prognostic biomarker development; prospective utility not established Code: No; data: No |
| Albalawi et al., 2025, Pakistan (61) | Public + private; BINO Hospital; TCGA-BRCA; Camelyon17; Multi-center; patients: 2847; slides/images: Internal 2,847 cases; external cohort sizes reported inconsistently within the article; tiles/annotations: 256×256 WSI patches | Surgical resection; H&E; Whole-slide image; timing: Primary surgery Clinical endpoint: Disease-free survival by AI-derived TIL density; | Foundation embeddings + graph spatial attention + dilated ResNet-50 + YOLO/U-Net detection-segmentation; learning: Supervised; pretraining: Foundation-model embeddings Explainability: Attention maps plus spatial TIL features | Internal and two claimed external cohorts plus deployment-style pilot; external: Yes; split: Patient/slide AUROC; accuracy; hazard ratio: Internal AUROC 0.92; external AUROCs 0.895 and 0.882; DFS HR 0.642; pathologist correlation r=0.879 | Risk stratification/prognostic biomarker development; prospective utility not established Code: No; data: No Overlap: Potential public-cohort reuse |
| Treatment response prediction |  |  |  |  |  |
| Ali et al., 2016, UK (62) | Private/internal; Neo-tAnGo trial; Multi-center trial; patients: 768; slides/images: Analysis sets varied: 614 pretreatment, 553 paired pre/post, 406 multivariable; tiles/annotations: Single-cell classification across digitized tissue sections | Core biopsy and surgical resection; H&E; Digitized diagnostic biopsy and surgical-resection slides; timing: Pre-treatment and post-treatment Clinical endpoint: pCR after neoadjuvant chemotherapy; | Single-cell classifier with absolute counts, fractions and neighbourhood-density metrics;  Explainability: Explicit spatial/cell/tissue features or maps | Internal trial-cohort modelling; subsequent independent validation reported separately in Ali 2017; external: No; split: Patient Odds ratio: Pretreatment lymphocyte density OR 4.46 (2.34–8.50) unadjusted and 2.42 (1.08–5.40) adjusted; post-treatment change OR 0.28 (0.17–0.47) | Pretreatment response stratification: prospective decision impact not established Code: Yes; data: No Overlap: Ali 2016 developed the biomarker; Ali 2017 independently validated it in ARTemis. |
| Ali et al., 2017, UK/Canada (63) Computational | Private/internal; ARTemis trial; Multi-center phase III trial; patients: 800; slides/images: 781 primary trial analysis; 609 baseline computational measures; 383 paired pre/post; tiles/annotations: Single-cell classification across digitized tissue sections | Core biopsy and surgical resection; H&E; Digitized diagnostic biopsy and surgical-resection slides; timing: Pre-treatment and post-treatment Clinical endpoint: Absence of invasive breast tumour and nodal disease after neoadjuvant chemotherapy; | Validated single-cell lymphocyte-density pipeline developed in Ali 2016;  Explainability: Explicit spatial/cell/tissue features or maps | Independent trial validation of the previously developed computational biomarker; external: Yes; split: Patient Odds ratio: Pretreatment lymphocyte density OR 2.92 unadjusted and 2.13 adjusted; paired-change adjusted OR 0.10 | Pretreatment response stratification: prospective decision impact not established Code: Yes; data: No Overlap: Ali 2016 developed the biomarker; Ali 2017 independently validated it in ARTemis. |
| Abe et al., 2019, Japan (64) | Private/internal; Single-center; patients: 105; slides/images: 105 core needle biopsy cases; tiles/annotations: Digital TIL density per mm2 stromal tissue | Biopsy; H&E; Whole-slide image; timing: Core needle biopsy before neoadjuvant anti-HER2 therapy Clinical endpoint: Pathological complete response to neoadjuvant anti-HER2 therapy; | Machine-learning-based classification of epithelial and stromal elements with digital TIL density measurement; Explainability: Handcrafted features with interpretable feature definitions | Internal split only; external: No; split: Patient OR: High TIL density (>2420/mm2) independently associated with pCR (P<0.0001) | Pretreatment response stratification: prospective decision impact not established Code: No; data: No |
| Li et al., 2021, China (65) | Private/internal; Single center/unclear; patients: 540; tiles/annotations: WSI tiles from tumor epithelium | Biopsy; H&E; Whole-slide image; timing: Pre-treatment biopsy Clinical endpoint: Pathological complete response to NAC; | DL tumor epithelium identification and pCR-score biomarker; | Internal validation; external: No; split: Patient AUC; OR; F1: pCR-score AUC 0.847 direct; logistic regression AUC 0.822; integrated model mean AUC 0.890 | Pretreatment response stratification: prospective decision impact not established Code: No; data: No |
| Li et al., 2022, China (66) | Private/internal; multi-center; patients: 874; tiles/annotations: WSI patches/tiles | Core needle biopsy; H&E; Whole-slide image; timing: Pre-treatment biopsy Clinical endpoint: Pathological complete response to neoadjuvant chemotherapy; | Deep learning pathological model (DLPM) from biopsy WSI features; Explainability: Not reported or limited | Internal + external validation; external: Yes; split: Patient AUC: DLPM AUC 0.72 in primary cohort; best external validation AUC 0.71; DLPM + clinical + sTILs AUC 0.82/0.80 in primary/validation cohort | Pretreatment response stratification: prospective decision impact not established Code: Yes; data: No |
| Li et al., 2022, China (67). | Private/internal; multi-center; tiles/annotations: WSI tiles from tumor-associated stroma | Biopsy; H&E; Whole-slide image; timing: Pre-treatment biopsy Clinical endpoint: Pathological complete response to NAC; | Deep learning tumor-associated stroma score (TS-score); Explainability: Explicit spatial/cell/tissue features or maps | Internal + external validation; external: Yes; split: Patient AUC; OR: TS-score AUC 0.729 in primary cohort; external validation AUCs 0.745, 0.673, and 0.725 | Pretreatment response stratification: prospective decision impact not established Code: Yes; data: No |
| Aswolinskiy et al., 2023, Netherlands/Italy (68) | Private + public; IMPRESS; BCSS, Multi-center; patients: 721 development/evaluation + 126 external validation; tiles/annotations: WSI segmentation outputs and biomarkers | Core needle biopsy; H&E; Whole-slide image; timing: Pre-treatment biopsy Clinical endpoint: Pathological complete response to NAC; | Deep-learning segmentation/detection of tumor, lymphocytes, stroma, mitoses; computational biomarkers; Explainability: Explicit spatial/cell/tissue features or maps | Internal + external validation; external: Yes; split: Patient AUC: AUC 0.66-0.88 across tested cohorts; external cTILs/LTR/ITR/MTR AUCs reported with 95% CIs | Pretreatment response stratification: prospective decision impact not established Code: Yes; weights: Partial; data: Yes |
| Choi et al., 2023, South Korea/USA (69) | Private/internal; Multi-center; patients: 402; slides/images: 402; tiles/annotations: Not applicable | Surgical resection and pre-NAC biopsy; H&E; Whole-slide image; timing: Mixed; pre-treatment biopsy for response cohort Clinical endpoint: Responder versus non-responder to neoadjuvant chemotherapy; | DL-based sTIL analyzer / Faster R-CNN-like cell detection implementation; | External validation dataset; external: Yes; split: Patient/slide CCC; OR: CCC 0.755 (95% CI 0.693-0.805); DL-assisted sTIL-high associated with response OR 1.28 (95% CI 1.01-1.63) | Pretreatment response stratification: prospective decision impact not established Code: Yes; data: Yes |
| Bhattarai et al., 2024, USA/Norway (70) | Private/internal; Single-center/unclear; patients: 76; slides/images: WSI triplets per patient; tiles/annotations: Hotspot image patches | Core needle biopsy; H&E + IHC; Whole-slide image; timing: Pre-treatment biopsy Clinical endpoint: pCR versus residual disease after NAC; | Mask R-CNN cell detection plus supervised ML classifiers for NAC response; | Internal cross-validation; external: No; split: Patient Accuracy; AUC: Top performance from combined tTIL, sTIL, tumor, Ki67+ and pH3+ hotspot features; exact value NR | Pretreatment response stratification: prospective decision impact not established Code: No; data: No |
| Fisher et al., 2024, USA/India/Europe (71) | Private/internal; multi-center; patients: 85 development + 79 validation; tiles/annotations: 468043 | Treatment-naive biopsy; H&E; Whole-slide image; timing: Pre-treatment biopsy Clinical endpoint: pCR versus residual disease after NAC; | Tile-level histology classifier; graph-based TME features; rbfSVM patient-level response classifier; Explainability: Explicit spatial/cell/tissue features or maps | Internal + external validation; external: Yes; split: Patient Accuracy: Tile validation accuracy 83.59%; NAC response prediction accuracy 81.01% in independent validation cohort | Pretreatment response stratification: prospective decision impact not established Code: No; data: No |
| Rasic et al., 2025, Denmark/Sweden (72) | Private/internal; multi-center; patients: 273 discovery + 245 validation; tiles/annotations: WSI segmentation/cell detection outputs | Core needle biopsy; H&E; Whole-slide image; timing: Pre-treatment biopsy Clinical endpoint: pCR after NACT; disease-free and overall survival; | Invasive cancer segmentation model plus fine-tuned multiclass StarDist for TIL quantification; learning: Supervised; pretraining: Histology model Explainability: Explicit spatial/cell/tissue features or maps | Internal + external validation; external: Yes; split: Patient Spearman correlation; OR; AUC; survival HR: AI-human Spearman R=0.61-0.77; baseline AUC 0.71; AI sTIL model AUC 0.76; ORs and 95% CIs reported | Pretreatment response stratification: prospective decision impact not established Code: No; weights: Yes; data: Yes |
| Liang et al., 2026, China (73) | Private/internal; Single-center response cohort with separate model-development data; patients: 32; slides/images: 32 pretreatment response-cohort images; additional specimens for TLS and cell-model development; tiles/annotations: TLS patches 5,357/2,770 at 3× and 3,963/2,565 at 10×; 21,889 lymphocytes and 19,208 non-lymphoid cells | Pretreatment core biopsy and development specimens; H&E; Whole-slide images; timing: Pretreatment Clinical endpoint: Miller–Payne response grade after neoadjuvant chemo-immunotherapy; | U-Net/MobileNetV2 TLS model, StarDist nuclei segmentation, MobileNetV2 cell classifier and 500-tree random forest response model; learning: Supervised; pretraining: Pretrained CNN components Explainability: Explicit spatial/cell/tissue features or maps | Independent technical TLS test set; response model repeated stratified five-fold cross-validation; external: No; split: Patient/image AUROC; accuracy; Cohen κ: Response AUROC 0.81 (0.76–0.86); TIL classifier accuracy 0.92; TLS test accuracy 0.87, κ=0.73 | Pretreatment response stratification: prospective decision impact not established Code: No; data: Yes |
| Schlam et al., 2026, USA/UK/Belgium/Australia (74) | Private clinical trial; NSABP B-41; Multi-center phase III trial; patients: 262; slides/images: 262 pretreatment H&E slides; 231 with gene-expression profiling; tiles/annotations: Single-cell and spatial features across WSIs | Core biopsy; H&E; Pretreatment whole-slide images; timing: Pretreatment Clinical endpoint: Trial pCR and event-free survival; | Zero-shot Case45 single-cell classifier with AI-TIL and tumour–immune spatial metrics; learning: Pretrained supervised model applied zero-shot; pretraining: External histopathology training data Explainability: Explicit spatial/cell/tissue features or maps | Trial-cohort analysis with internal modelling; no separate external clinical validation; external: No; split: Patient AUROC; hazard ratio: Best pCR model AUROC 0.685; spatial + gene-expression model AUROC 0.701; AI-TIL EFS HR 0.52 (0.29–0.94) | Pretreatment response stratification: prospective decision impact not established Code: No; data: No |
| Xie et al., 2026, China (75) | Private/internal; Multi-center; patients: 131; slides/images: Pretreatment H&E WSIs from 96 discovery and two validation cohorts (18 and 17 patients); tiles/annotations: Cell-level QuPath classifier and spatial clustering outputs | Biopsy; H&E; Whole-slide image; timing: Pre-treatment core needle biopsy Clinical endpoint: Response to neoadjuvant chemotherapy and survival outcomes by TIL spatial subtype; | QuPath cell classifier and AI-powered spatial clustering of focal-hotspot/diffuse immune subtypes; Explainability: Explicit spatial/cell/tissue features or maps | Discovery cohort plus two independent external validation cohorts; external: Yes; split: Patient OR: Spatial TIL subtypes associated with HER2 expression, pCR and survival; multivariable models attenuated pCR independence | Pretreatment response stratification: prospective decision impact not established Code: No; data: No |

Abbreviations: AI, artificial intelligence; AUC, area under the curve; AUROC, area under the receiver operating characteristic curve; CCC, concordance correlation coefficient; CD3/CD4/CD8/CD20/CD68, cluster of differentiation markers 3/4/8/20/68; CI, confidence interval; CNN, convolutional neural network; C-index, concordance index; DCIS, ductal carcinoma in situ; DFS, disease-free survival; DL, deep learning; DLPM, deep learning pathological model; EFS, event-free survival; ER, estrogen receptor; FOXP3, forkhead box P3; FROC, free-response receiver operating characteristic curve; GUI, graphical user interface; H&E, hematoxylin and eosin; HER2, human epidermal growth factor receptor 2; HR, hazard ratio; IHC, immunohistochemistry; MIL, multiple-instance learning; mpp, microns per pixel; NAC, neoadjuvant chemotherapy; NR, not reported or not extracted; OR, odds ratio; OS, overall survival; pCR, pathological complete response; PD-L1, programmed death-ligand 1; PFI, progression-free interval; RF, random forest; RFS, relapse-free survival; ROI, region of interest; SSL, self-supervised learning; SVM, support vector machine; sTIL, stromal tumor-infiltrating lymphocyte; TIL, tumor-infiltrating lymphocyte; TLS, tertiary lymphoid structure; TMA, tissue microarray; TME, tumor microenvironment; TNBC, triple-negative breast cancer; WSI, whole-slide image; YOLO, you only look once..
